# System-Wide Adaptations of *Desulfovibrio alaskensis* G20 to Phosphate-Limited Conditions

**DOI:** 10.1371/journal.pone.0168719

**Published:** 2016-12-28

**Authors:** Tanja Bosak, Florence Schubotz, Ana de Santiago-Torio, Jennifer V. Kuehl, Hans K. Carlson, Nicki Watson, Mirna Daye, Roger E. Summons, Adam P. Arkin, Adam M. Deutschbauer

**Affiliations:** 1 Department of Earth and Planetary Science, Massachusetts Institute of Technology, Cambridge, Massachusetts, United States of America; 2 University of Bremen and MARUM, Bremen, Germany; 3 Environmental Genomics and Systems Biology Division, Lawrence Berkeley National Laboratory, Berkeley, California, United States of America; 4 W.M. Keck Microscopy Facility, The Whitehead Institute, Cambridge, Massachusetts, United States of America; 5 Department of Bioengineering, University of California, Berkeley, Berkeley, California, United States of America; Universite Paris-Sud, FRANCE

## Abstract

The prevalence of lipids devoid of phosphorus suggests that the availability of phosphorus limits microbial growth and activity in many anoxic, stratified environments. To better understand the response of anaerobic bacteria to phosphate limitation and starvation, this study combines microscopic and lipid analyses with the measurements of fitness of pooled barcoded transposon mutants of the model sulfate reducing bacterium *Desulfovibrio alaskensis* G20. Phosphate-limited G20 has lower growth rates and replaces more than 90% of its membrane phospholipids by a mixture of monoglycosyl diacylglycerol (MGDG), glycuronic acid diacylglycerol (GADG) and ornithine lipids, lacks polyphosphate granules, and synthesizes other cellular inclusions. Analyses of pooled and individual mutants reveal the importance of the high-affinity phosphate transport system (the Pst system), PhoR, and glycolipid and ornithine lipid synthases during phosphate limitation. The phosphate-dependent synthesis of MGDG in G20 and the widespread occurrence of the MGDG/GADG synthase among sulfate reducing ∂-Proteobacteria implicate these microbes in the production of abundant MGDG in anaerobic environments where the concentrations of phosphate are lower than 10 μM. Numerous predicted changes in the composition of the cell envelope and systems involved in transport, maintenance of cytoplasmic redox potential, central metabolism and regulatory pathways also suggest an impact of phosphate limitation on the susceptibility of sulfate reducing bacteria to other anthropogenic or environmental stresses.

## Introduction

Sulfate reducing microbes couple the oxidation of organic matter or hydrogen to the reduction of sulfate and link the cycles of sulfur, carbon and oxygen in anaerobic marine environments and sulfate-rich lakes. Studies of microbial sulfate reduction have focused primarily on the energy conservation, reduction of heavy metals, the degradation of particular organic substrates, and the geochemical signals of this process in the environment [1, 2], as well as the responses of *Desulfovibrio vulgaris* Hildenborough to biocides, oxygen, nitrite, high temperature and pH stress [3–9]. Currently, much less is known about adaptations and responses of sulfate reducing bacteria to environmental limitations other than the lack of sulfate or electron donors [10].

The low availability of phosphate, a key reactant in various biosynthetic, metabolic and regulatory pathways, is thought to limit the cell growth and primary productivity in marine surface waters [11, 12]. To reduce the cellular requirement for phosphate in oxygenated soils and waters, some marine cyanobacteria and aerobic heterotrophic bacteria, and some soil bacteria synthesize glycolipids, amino lipids or teichuronic acids all of which are devoid of phosphorus [13–19]. Recent studies have shown that up to 80% of the total polar lipids in suboxic and anaerobic environments including the Black Sea, Labrador Sea and Baltic Sea do not contain phosphorus [20–22], perhaps in response to phosphate limitation. This stoichiometric signal is unexpected, because organic degradation and the microbial cycling of nitrogen in suboxic and anoxic marine waters and sediments are thought to increase the P:C and the P:N ratios [23–25]. These processes increase the concentrations of phosphate to 4–10 μM in the water column or 80 μM in sediments [26], i.e., orders of magnitude above the concentrations in oxygenated surface oceans. Yet, given that similar or even higher concentrations of phosphate induce phosphate limitation in cultures of some aerobic heterotrophic Proteobacteria (e.g., [13, 27, 28]), adaptations of anaerobic marine microbes to phosphate limitation warrant a closer look.

System-level studies of relevant model organisms can improve our ability to recognize and interpret signals of environmental phosphate limitation, particularly of sulfate reducing ∂-Proteobacteria of the genera *Desulfovibrio*, *Desulfosarcina/Desulfococcus* and *Desulfobacterium* along with other known inhabitants of suboxic and sulfidic marine sediments and the water column [29–33]. To date, only few such reports exist [34, 35], and they do not address the composition of polar lipids, a geochemical parameter that is commonly used to characterize the microbial diversity and processes in environmental samples. To bridge this gap, this study identifies genes important for fitness during phosphate-limited growth and the survival after phosphate starvation of the model sulfate reducing ∂-Proteobacterium *Desulfovibrio alaskensis* G20 using barcoded transposon mutants. Microscopic, chemical and lipid analyses of wild-type cells and individual gene mutants grown at environmentally relevant phosphate concentrations further characterize mechanisms by which G20 adapts to phosphate limitation and shore up the evidence for widespread phosphate limitation in suboxic and anaerobic marine environments.

## Methods

### Strains and culture conditions

We used the wild-type G20 cured of a plasmid from the strain collection in the Deutschbauer laboratory. The barcoded transposon mutant pools and individual transposon mutant strains used in this study were previously described [36]. Wild-type G20, the mutant pools, and the individual transposon mutants were grown in batch cultures within an anaerobic chamber with an atmosphere composed of 90:5:5% N_2_, CO_2_ and H_2_. The growth temperature for experiments was 30°C, unless stated otherwise. All glassware was rinsed and autoclaved with nanopure water three times to remove any adsorbed phosphate. Hungate tubes were closed by butyl rubber stoppers with aluminum seals.

All strains grew in MOLS4 medium [37] with modified concentrations of phosphate (see descriptions of experiments below for details). Basal MOLS4 (pH 7.2) contained 60 mM sodium lactate, 30 mM sodium sulfate, 8 mM magnesium chloride, 20 mM ammonium chloride, 2 mM potassium chloride, 0.6 mM calcium chloride, 30 mM Tris-HCl buffer (pH 7.4), iron(II) chloride-EDTA (0.06 mM), 6 ml/L of trace element solution and 1 ml/L of Thauer’s vitamin solution [38]. The trace element solution was prepared in 10% (v/v) HCl and contained per 1 liter; 1.5 g FeCl_2_, 4H_2_O; 0.20 g CoCl_2_, 6H_2_O; 0.1 g MnCl_2_, 4H_2_O; 70 mg ZnCl_2_; 8 mg H_3_BO_3_; 40 mg Na_2_MoO_4_, 2H_2_O; 25 mg NiCl_2_, 6H2O; 10 mg CuCl_2_, 2H_2_O; 8 mg Na_2_SeO_3_; 15 mg Na_2_WO_4_, 2H_2_O; 10 mg V_2_O_5_. MOLS4 was boiled and flushed with anaerobic gas, autoclaved and transferred to the anaerobic chamber while still warm. Sterile medium was reduced before inoculation by the addition of 1 mM Na_2_S from a sterile, anaerobic 1 M stock solution.

All individual strains and mutant pools were recovered from frozen stocks by growth in MOLS4 medium with 0.5 mM K_2_HPO_4_ and 0.1% yeast extract (rich medium). Recovered cells were harvested in mid-log phase, washed three times by anaerobic centrifugation at room temperature in phosphate-free MOLS4 lacking yeast extract and inoculated into basal MOLS4 to the initial OD_600_ value of 0.02. To achieve desired concentrations of phosphate in the cultures we added sterile, anaerobic K_2_HPO_4_ to a final concentration of 2, 10 or 500 μM. To establish comparable concentrations of potassium in all cultures, we added 0.5 mM KCl from a sterile 1 M stock solution to cultures containing 0, 2 and 10 μM K_2_HPO_4_. The growth was monitored by measurements of optical density at 600 nm (OD_600_) using a ThermoScientific Spectronic 20D+ spectrophotometer or by measuring the OD_600_ values of 150 μl subsamples on a Synergy 2 Multi-Mode microplate Reader (BioTek, Winooski, VT). To adapt cells to low phosphate concentrations, we grew them in media with the desired low phosphate concentrations as described above and transferred these vegetatively growing cells at 5% v/v into media with the same initial concentration of phosphate. All analyses were conducted on samples harvested during vegetative growth and repeated twice. Sulfide concentrations were measured colorimetrically as described previously [39, 40].

Transposon insertions in Dde_3613, Dde_3661 and the wild-type G20 strain were analyzed by transmission electron and epifluorescence microscopy after growth in MOLS4 in 100 ml triplicate cultures with a 100% N_2_ atmosphere at 27°C after one wash and two transfers, as described above. To establish more comparable culture conditions between these experiments and our previous studies of sulfate-reducing bacteria [40, 41], we reduced the high concentrations of sodium lactate and sodium sulfate from the original MOLS4 recipe (see above) to 20 and 21 mM, respectively. This did not change the growth rates or yields of phosphate-limited cultures, but it limited the supply of the electron donor in cultures containing 200 μM phosphate or more.

### Fitness assays of pooled mutants

All fitness assays used two pools of G20 transposon mutants as described previously [36, 42] and quantified the abundances of uniquely tagged strains that carry transposon deletions in different genes under phosphate-limited conditions of interest, as well as in control, phosphate-replete conditions. Pool 1 contained 4,069 unique strains and Pool 2 contained 4056 unique strains [43]. The G20 transposon mutants contain TagModules, or unique DNA barcodes, that serve as unique strain identifiers that can be quantified in parallel by microarray hybridization or DNA barcode sequencing. Together, the two pools of mutants probed the fitness of 2,338 out of 3528 unique protein-coding genes in the genome of G20 (66%). If a gene has negative score (fitness defect), mutants lacking this gene grow less well in the tested condition relative to the control condition. Conversely, a gene will have a positive fitness score (fitness benefit) if mutants lacking this gene grow better in the tested condition relative to the control condition.

The two pools of mutants were recovered from frozen stocks in rich media, washed and grown separately to mid-logarithmic phase (OD_600_ ~ 0.6) in MOLS4 containing 500 μM phosphate (phosphate-replete condition) without yeast extract. These cells were harvested as the “start” control for all fitness assays. Additional aliquots of the two recovered pools were washed three times by centrifugation at 14,000 rpm for 2 min and resuspension in MOLS4 without phosphate. The washed cells were inoculated into cultures for fitness assays at initial OD_600_ values of 0.02.

Fitness assays of pooled *D*. *alaskensis* G20 mutants employed two different approaches: the first probed fitness during phosphate-limited growth, the second one at multiple time points during phosphate starvation. Because the final cell densities in phosphate-limited cultures were low and only a few doublings occurred, we transferred and regrew the cultures twice to increase the number of population doublings. Briefly, G20 pools grew with 10 μM initial phosphate (limiting concentration) until their OD_600_ values were 0.2. One-ml aliquots from these cultures were transferred into two separate Hungate tubes containing 9 mL of sterile medium with 10 μM phosphate. This was repeated one more time, and the entire 10 ml culture volumes of the two mutant pools were then collected for fitness assays. The OD_600_ value was 0.21 at the time of collection, compared to the maximum OD_600_ value of 0.35 for the wild type cultures after two transfers into MOLS4 with 10 μM initial phosphate. Cells were pelleted by centrifugation at 8,000 rpm at 4°C for 8 minutes and stored at -20°C until further analyses (DNA extraction, quantification of the abundances of different uniquely tagged strains).

A different assay measured the abilities of different mutants to survive phosphate starvation and resume growth upon encountering nutrient-replete conditions. We grew Pools 1 and 2 separately in 9 ml of the medium containing 500, 10 or 0 μM phosphate and sampled 1 ml of each culture 5, 10 and 15 days after the onset of stationary phase, as determined from the measurements of OD_600_ values. The final OD_600_ values in cultures with 500, 10 and 0 μM initial phosphate, respectively, were 0.8, 0.26 and 0.17, respectively. To recover cells after starvation and obtain visible cell pellets, we inoculated the sampled 1-ml aliquots of stationary phase cultures into 9 ml of MOLS4 with 500 μM phosphate and yeast extract (rich medium). Cells from cultures with 500 μM phosphate attained OD_600_ values of 0.8, i.e., the maximum OD value in two days. Those from cultures with 0 and 10 μM initial phosphate took three or more days to reach the same OD_600_ value. This suggested that more cells had died or become non-viable during phosphate starvation. The recovered mutant pools in late exponential or early stationary phase (OD_600_ value 0.8) were harvested by anaerobic centrifugation at 14,000 rpm for 2 min at room temperature and stored at -20°C until further analyses (DNA extraction, quantification of the abundances of different uniquely tagged strains).

We extracted genomic DNA from all samples and used PCR to amplify the DNA barcodes that uniquely identify mutant strains [43, 44]. Previous studies of G20 mutant pools mixed the PCR reactions with amplified “uptags” and “downtags” from each sample and hybridized them to an Affymetrix 16K TAG4 microarray [36, 42–45]. However, for the present work, we sequenced amplified “uptags” on an Illumina MiSeq using a BarSeq method [46]. For BarSeq, gene fitness values were calculated as described in Wetmore et al. [46].

The fitness value for a gene was calculated as the average of fitness values for all relevant strains with insertions in that gene from both pools, as previously described [36, 42]. The reported data present only the averaged gene fitness values normalized to a zero-density distribution [42]. The use of both pools provided internal replicates and a test of internal consistency, because 1091 strains were present in both pools. Each gene fitness value reported in this paper was measured as the ratio of the gene fitness values for “start” cultures relative to gene fitness values at the end of the experiment, log_2_(start/end).

We filtered the original data set (S1 and S4 Tables) to remove genes with fitness scores that varied little across the seven probed conditions (standard deviation < 0.3) and were thus unlikely to be important for fitness under conditions of interest. The remaining genes were identified as important for fitness under our experimental conditions if: 1. their fitness scores in both phosphate-starved cultures (with 0 or 10 μM added phosphate) differed by more than 0.95 units from the score in the corresponding phosphate-replete control condition, or if 2. the fitness score in the vegetatively growing culture (with initial 10 μM phosphate) differed by more than 0.95 units from the score in the starting control culture. This corresponded to 1.93-fold (2^0.95^) increases or decreases of the relevant mutants relative to their abundances in the control conditions. Next, we excluded genes important for fitness in all conditions: the absolute values of their scores were greater than 0.95, but the scores were not significantly different (> = 0.95 units) between the vegetatively growing culture and all cultures in stationary phase. We also excluded genes important for fitness during stationary phase (|score| > 0.95), but not in specific response to phosphate; their scores were not more than 0.95 units different in both phosphate starved cultures and the corresponding control condition.

The fitness patterns of genes identified in this manner were analyzed as a function of experimental conditions in Multiple Expression Viewer (MEV by TIGR) [47]. Genes and conditions with similar patterns of normalized fitness scores [48] among the seven tested conditions were identified by hierarchical clustering analysis (HCL in MEV), optimizing the gene leaf order and the sample leaf order. The nine clusters of important genes (Table 1, S1 Fig) contained genes with fitness scores with linear (Pearson) correlation coefficients higher than 0.62 across all seven experimental conditions. We also used MEV to compare the fitness scores of important genes measured in our study to the fitness scores measured for the same genes under different growth and stress conditions. The results of previous experiments were downloaded from the microbesonline.org database [36, 49–51]. We used OperonDB http://operondb.cbcb.umd.edu/cgi-bin/operondb/pairs.cgi?genome_id=329 and microbesonline.org to determine whether genes occurred in the same operon.

**Table 1:** Fitness scores of genes important during phosphate-limited growth and for the survival after phosphate starvation.

Gene ID	Product	5d, 0	5d, 10	5d, 500	10d, 0	10d, 10	10d, 500	Veg. 10	Function
**Cluster I**									
Dde_3134	adenosylhomocysteinase	-2.22	-3.02	-2.73	-3.11	-3.68	-2.65	-4.68	amino acid biosynthesis^&^^,^^#^
Dde_1061	PstC	-0.40	-0.58	-0.28	-0.50	-0.92	-0.05	-1.49	*PstC*, *phosphate transport*
Dde_2386	PstB	-0.78	-0.69	-0.15	-0.77	-1.41	0.28	-3.09	*PstB*, *phosphate transport*
Dde_1060	PstA	-0.57	-0.73	-0.29	-0.76	-1.64	0.06	-3.74	*PstA*, *phosphate transport*
Dde_1062	PstS	-0.40	-0.65	-0.34	-0.87	-1.69	-0.03	-3.81	*PstS*, *phosphate uptake*
Dde_1447	dephospho-CoA kinase	-0.11	-0.12	-0.14	-0.85	-0.95	-0.25	-4.71	CoA biosynthesis
Dde_3661	putative ornithine lipid synthase	-0.07	-0.09	0.02	-0.91	-0.39	0.27	-1.56	OlsF homolog, ornithine lipid biosynthesis, cell envelope biosynthesis
Dde_1329	ABC-type dipeptide transport system, periplasmic component	-0.92	-1.33	-0.51	-2.86	-2.01	-0.86	-3.05	transport
Dde_2210	permease component of zinc ABC transporter	-1.68	-2.43	-0.99	-3.93	-3.72	-1.61	-5.30	transport
Dde_2201	geranyltranstransferase	-2.42	-2.24	-1.57	-2.81	-2.13	-1.25	-2.86	lipid biosynthesis
Dde_3782	multi-sensor signal transduction histidine kinase	-1.84	-1.47	-0.19	-1.55	-1.44	0.10	-2.51	*PhoR homolog*, *phosphate metabolism*
Dde_3613	glycosyltransferase group I	-0.96	-0.47	0.13	-0.45	-0.05	0.22	-1.29	Agt homolog, glycolipid biosynthesis, cell envelope biosynthesis
**Cluster II**									
Dde_0362	Sugar transferase	-0.93	-0.88	-0.1	-0.95	-1.09	-0.46	-0.95	cell envelope biogenesis
Dde_0652	HmcB, 40.1 kd protein in hmc operon	0.08	0.12	1.36	0.07	0.12	1.31	0.41	electron transfer
Dde_0649	HmcE, 25.3 kd protein in hmc operon	0.06	0.13	1.50	0.04	0.05	1.39	0.43	electron transfer
Dde_0653	HmcA, high molecular weight cytochrome c	-0.56	-0.39	0.97	-0.71	-0.76	1.00	0.01	electron transfer
Dde_3385	hypothetical protein	-1.23	-1.03	-0.70	-1.51	-1.68	-0.39	-1.41	unknown
Dde_3255	UDP-N-acetylglucosamine 2-epimerase	-1.58	-0.91	-0.57	-1.71	-1.58	-0.21	-0.97	polysaccharide biosynthesis, cell envelope biosynthesis
Dde_3008	hypothetical protein	-1.52	-1.20	-0.93	-1.78	-1.80	-0.37	-1.12	unknown
Dde_1565	ABC-type dipeptide transport system, periplasmic component	-1.16	-0.81	-0.64	-1.43	-1.49	-0.26	-0.94	transport
Dde_2301	VacJ family surface lipoprotein	-1.35	-0.89	-0.79	-1.80	-1.62	-0.26	-1.09	cell envelope
Dde_2299	MlaD homolog	-1.33	-1.30	-0.62	-1.63	-1.92	-0.49	-1.09	cell envelope
Dde_3561	methyl-accepting chemotaxis protein	-1.26	-0.90	-0.39	-1.37	-1.60	-0.26	-0.81	signaling, chemotaxis
Dde_2298	ATPase, MlaF homolog	-1.25	-0.94	-0.77	-1.48	-1.82	-0.46	-1.03	phospholipid transport, MlaF homolog, cell envelope
Dde_2300	toluene tolerance family protein	-1.13	-0.81	-0.56	-1.49	-1.70	-0.29	-0.81	unknown
Dde_0534	putative transposase protein	-1.18	-0.99	-0.50	-1.71	-1.93	-0.45	-0.92	nucleic acid processing and recombination
Dde_3092	heat shock protein, class I, Hsp20	-1.61	-1.44	-1.19	-2.21	-2.41	-0.96	-1.48	stress response
Dde_2297	Orf, hypothetical protein	-1.38	-1.19	-0.86	-2.34	-2.76	-0.68	-1.39	unknown
Dde_1246	type 11 methyltransferase	-1.16	-1.03	-0.85	-1.74	-1.71	-0.57	-1.20	unknown
Dde_2655	biotin synthase	-1.11	-0.93	-1.04	-1.52	-1.62	-0.25	-1.05	vitamin biosynthesis
Dde_0341	ATP-dependent RNA helicase DeaD (deaD)	-0.26	-0.34	0.07	-0.38	-0.52	0.62	-0.14	RNA processing
Dde_2366	Flp pilus assembly protein TadD, contains TPR repeats	-0.78	-0.25	0.17	-0.24	-0.65	0.96	0.2	pilus assembly^&^^,^^^^
**Cluster III**									
Dde_1684	nitrogen-specific histidine kinase NtrB	-1.49	-1.30	-0.2	-0.52	-0.54	-0.40	-0.42	nitrogen metabolism
Dde_2945	phosphomannomutase/ phosphoglucomutase	-3.10	-3.12	-2.03	-2.73	-2.60	-2.07	-2.30	cell envelope biosynthesis
**Cluster IV**									
Dde_1023	molecular chaperone DnaK	-1.06	-1.42	-1.54	-3.13	-3.39	-1.94	-2.70	recombination and regulation^&^
Dde_2285	1,4-alpha-glucan branching enzyme	-0.26	-0.14	0.08	-2.64	-3.00	-0.76	-1.35	polysaccharide metabolism
Dde_1781	RNA metabolizing metallo-beta-lactamase	0.01	-0.09	0.01	-1.56	-0.94	0.87	-0.78	RNA processing
Dde_3232	hypothetical protein	-0.57	-0.51	-0.32	-1.28	-1.23	-0.25	-0.94	unknown
Dde_0774	sensor histidine kinase/response regulator	-2.34	-1.58	-0.85	-5.08	-3.13	-0.88	-3.07	signaling and sensing, CheY-like
Dde_2555	hypothetical protein	-1.43	-0.95	-0.24	-3.23	-1.92	-0.27	-1.89	unknown
Dde_0359	sugar O-acyltransferase, NeuD family	-1.54	-1.96	-1.01	-3.58	-2.79	-1.50	-3.07	cell envelope biosynthesis
Dde_0014	methionyl-tRNA formyltransferase	-2.63	-3.56	-2.85	-4.24	-4.29	-2.14	-3.15	folate metabolism, peptide biosynthesis, amino acid metabolism^&^
Dde_0572	carboxynorspermidine synthase	-2.38	-3.04	-2.59	-3.81	-3.76	-2.45	-2.79	polyamine biosynthesis^&^
Dde_3105	citrate-dependent iron(III) transport protein	-1.69	-1.18	-1.41	-2.11	-2.63	-1.07	-1.05	iron transport
**Cluster V**									
Dde_1569	(p)ppGpp synthetase II	-3.52	-3.24	-4.14	-1.97	-3.04	-4.77	-0.86	regulation^&^
Dde_1008	bifunctional histidinal dehydrogenase	-5.42	-5.60	-6.18	-4.75	-5.26	-6.22	-4.60	amino acid metabolism^#^
Dde_3719	BadM/Rrf2 family transcriptional regulator	-1.98	-1.88	-2.83	-0.48	-1.19	-3.84	-0.82	regulation
Dde_0979	Conserved hypothetical protein	-2.90	-3.43	-3.97	-2.51	-3.03	-4.80	-2.57	unknown
Dde_1106	5-enolpyruvylshikimate-3-phosphate synthase	-1.25	-1.33	-1.64	-1.04	-1.13	-2.41	-0.75	amino acid metabolism^#^
Dde_3717	response regulator containing CheY-like receiver	-0.64	-1.62	-2.11	-0.04	-0.31	-1.86	-0.55	regulation
Dde_3711	conserved hypothetical protein	-0.90	-1.32	-2.03	-0.14	-0.36	-1.85	-0.57	unknown
Dde_3718	multi-sensor signal transduction histidine kinase	-0.55	-0.87	-1.75	0.08	0.01	-1.42	-0.33	regulation, in operon with Dde_3717
Dde_0398	acetolactate synthase catalytic subunit	-1.22	-1.07	-2.07	-0.07	-0.31	-1.90	-0.63	amino acid metabolism^&^
Dde_3712	universal stress protein family	-1.03	-0.97	-1.67	0.01	-0.27	-1.56	-0.46	stress response
Dde_3713	UspA domain-containing protein	-1.19	-1.47	-1.98	-0.10	-0.27	-1.65	-0.63	stress response
Dde_1775	PTS system fructose transporter	-0.75	0.06	-1.00	0.50	0.38	-1.32	0.16	sugar transport
Dde_3715	multi-sensor signal transduction histidine kinase	0.12	0.03	-1.19	0.23	0.15	-0.88	-0.24	regulation
Dde_0480	O-antigen polymerase	0.38	0.27	-1.11	-0.22	0.39	-1.15	-0.21	cell envelope biosynthesis
Dde_3469	metallophosphoesterase	0.53	0.31	0.05	-0.05	-0.04	-1.09	-0.22	protein, lipid or nucleic acid processing
Dde_3450	DNA polymerase I	-2.43	-2.92	-3.45	-3.47	-3.59	-7.38	-3.36	replication
Dde_0537	ribonuclease E (rne)	0.00	-0.87	-2.09	-1.25	-1.26	-3.96	-1.01	RNA processing^&^
Dde_2512	transcription elongation factor GreA	0.34	0.15	-0.61	0.34	0.17	-1.04	0.13	transcription^#^
Dde_2672	hypothetical protein	-0.68	-0.57	-1.61	-0.61	-0.89	-2.22	-0.60	unknown^&^
Dde_1114	conserved hypothetical protein	-3.91	-3.62	-4.59	-3.67	-4.32	-5.97	-4.11	unknown^&^
Dde_2076	cytochrome B561	-0.59	-0.36	-0.68	-0.57	-0.31	-1.57	-0.67	electron transfer^&^
Dde_1175	RNA-binding protein	-0.68	-0.20	-1.34	-0.89	-0.57	-2.78	-0.68	unknown
Dde_2414	Hypothetical	-0.24	-0.03	-0.45	-0.25	-0.05	-1.32	0.12	unknown
Dde_1807	hypothetical	-0.63	-0.55	-0.72	-0.44	-0.46	-1.63	-0.38	B6 dependent amino acid metabolism^&^
Dde_1028	AsmA protein, putative	-0.28	-0.31	-0.71	-0.33	-0.30	-1.94	-0.05	cell envelope biosynthesis^&^
Dde_1806	apolipoprotein N-acyltransferase	0.10	0.15	-0.02	0.05	0.13	-1.48	0.22	lipoprotein biosynthesis, cell envelope biosynthesis
Dde_0249	GTP cyclohydrolase subunit MoaC	-0.70	-0.53	-0.54	-0.32	-0.17	-2.50	-0.57	molybdenum cofactor biosynthesis protein C^&^
Dde_0709	molybdopterin biosynthesis, protein A	-0.52	-0.37	-0.29	-0.26	-0.24	-2.16	-0.24	cofactor biosynthesis^&^
Dde_3228	molybdenum cofactor biosynthesis protein (moeA-1)	-0.62	-0.30	-0.32	-0.19	-0.13	-2.07	-0.09	Mo cofactor biosynthesis^&^^,^
Dde_1390	molybdenum cofactor synthesis domain-containing protein	-0.82	-0.45	-0.32	-0.44	-0.17	-2.01	-0.12	cofactor biosynthesis^&^
Dde_2944	4Fe-4S ferredoxin	-0.33	-0.10	-0.12	-0.39	-0.08	-1.64	-0.06	electron transfer
Dde_2943	aldehyde ferredoxin oxidoreductase (aor-2)	-0.38	-0.14	0.01	-0.44	-0.12	-1.61	-0.16	electron transfer
Dde_0230	molybdopterin biosynthesis	-0.89	-0.33	-0.22	-0.85	-0.26	-2.10	-0.37	cofactor biosynthesis^&^^,^
**Cluster VI**									
Dde_0382	ParA homolog ATPase	-4.18	-4.40	-3.93	-2.99	-2.38	-4.64	-4.81	chromosome partitioning, cell division^&^
Dde_3596	aspartate transaminase	-1.47	-2.32	-2.50	-1.55	-1.67	-2.91	-2.96	amino acid metabolism, oxoacid metabolism^#^
**Cluster VII**									
Dde_0043	ferredoxin-like protein	-0.94	-0.87	-0.12	0.14	0.05	-1.09	0.12	carbon metabolism^^^
**Cluster VIII**									
Dde_1261	integral membrane sensor hybrid histidine kinase	0.65	0.40	0.18	0.65	0.44	0.13	1.38	regulation^^^
Dde_1256	fumarate reductase, iron sulfur protein	0.61	0.20	0.11	0.62	0.28	0.00	1.13	carbon metabolism
Dde_1260	Fis family transcriptional regulator	0.68	0.36	0.23	0.69	0.41	0.03	1.29	regulation^^^
Dde_1258	Fumarate reductase respiratory complex	0.71	0.26	0.22	0.65	0.26	-0.05	1.33	carbon metabolism^^^
Dde_2673	ferrous iron transporter component feoA	-3.40	-5.45	-4.14	-4.18	-5.01	-6.04	-1.74	iron transport^&^^,^
Dde_1254	fumarate hydratase, class I	-0.11	-0.70	-0.17	-0.36	-0.51	-0.86	1.09	carbon metabolism
Dde_0153	hypothetical protein	0.07	0.04	0.08	-0.02	-0.01	0.06	2.52	unknown, in operon with Mo ABC transporter permease and a periplasmic Mo-binding protein
**Cluster IX**									
Dde_3201	cobyrinic acid ac-diamide synthase	-3.30	-5.04	-4.47	-5.88	-5.42	-6.71	-3.98	cofactor biosynthesis^&^^,^
Dde_3707	hypothetical	-0.11	-0.93	-2.28	-2.51	-3.77	-3.70	-1.93	unknown
Dde_3774	hypothetical	1.04	-0.87	-1.89	-1.60	-1.92	-2.39	-1.69	unknown
Dde_3773	hypothetical protein	-1.10	-2.19	-3.72	-2.85	-3.15	-4.27	-2.98	unknown

^&^Differences between phosphate replete and phosphate starved cultures decrease 15 days after the onset of stationary phase by more than 0.59 units or change sign in at least one phosphate starved culture.

^#^ Fitness defect increases by more than 0.59 units in phosphate replete culture 15 days after the onset of stationary phase.

^$^ Fitness defects increase in both phosphate starved cultures 15 days after the onset of stationary phase by more than 0.59 units.

^^^Fitness benefits increase in one or both phosphate starved culture by more than 0.59 units 15 days after the onset of stationary phase.

Blue shading identifies genes with predicted direct roles in phosphorus homeostasis, rose-colored shading identifies genes with predicted or confirmed direct roles in the biosynthesis of the cell envelope, yellow shading identifies genes with predicted roles in transport and green shading shows genes encoding the Hmc complex. The same color scheme identifies the same genes in Fig 3.

To verify the results of fitness assays with pooled mutants, we measured the growth of six individual mutant strains in phosphate-limited MOLS4 (Dde_3661, Dde_3613, Dde_2285, Dde_1023, Dde_3255 and Dde_1565, as underscored in Table 1) relative to wild-type G20. Two successive transfers of individual strains (see the sections *Strains and Culture Conditions* and *Fitness Assays of Pooled Mutants* above) into separate duplicate cultures of MOLS4 with 10 μM initial phosphate increased the number of doublings during phosphate-limited growth. The reported growth curves show OD_600_ values of duplicate vegetative cultures growing at 30°C after the second transfer and confirm the fitness defects for all strains (S2 Fig). Supporting Information contains measurements of OD600 in the cultures of wild-type G20 and various mutants.

### Lipid analyses

Wild-type G20, Dde_3613 and Dde_3661 were grown in duplicate 10 ml cultures (containing 500 μM initial phosphate) or 45 ml cultures (containing 10, 2 and 0 μM initial phosphate) as described in the section about *Strains and Culture Conditions*. The cells were harvested by centrifugation of 30-ml culture volumes at 9000 rpm at 4°C for 5 min. The addition of 5 ml of 50 mM Zn-acetate precipitated sulfide and facilitated the centrifugation. Pelleted cells were stored at -80°C before analyses. Cultures of Dde_3613 and Dde_3661 that lacked any added phosphate had very low biomass, so we were not able to analyze the lipids or measure growth in these cultures.

A modified Bligh-Dyer method [52] was used to extract lipids from cell pellets. The cell pellets were transferred into solvent-cleaned polytetrafluoroethylene tubes, amended with ca. 2 g of combusted sand and suspended in 20 ml of a solvent mixture consisting of 2:1:0.8 (v:v:v) methanol:dichloromethane(DCM):phosphate buffer solution (K_2_HPO_4_, 50 mM, pH 7.4). Two extraction steps were performed where the samples were ultrasonicated for 10 minutes, centrifuged for 10 min at 2500 rpm and the supernatant collected in a separatory funnel. To separate an aqueous and organic phase, we added 20 ml DCM and deionized water and washed each phase two times with DCM and deionized water, respectively. The lipid-containing organic phase was collected and evaporated to dryness under nitrogen gas. An aliquot of the total lipid extract was directly analyzed via high performance liquid chromatography mass spectrometry (HPLC-MS) using a Dionex Ultimate 3000RS UHPLC coupled to a Bruker maXis high resolution quadrupole time-of-flight (Q-TOF) mass spectrometer with an electrospray ionization interface (ESI) in positive ionization mode [53].

Intact polar lipids (IPLs) were separated by hydrophilic interaction (HILIC) chromatography using a Waters Acquity UPLC BEH Amide column (3.5 μm, 2.1 x 150 mm) after [53]. The solvent system was set from 99% A and 1% B to 5% B in 4 min and 25% B in 22.5 min and finally raised to 50% B in 26.5 min. These conditions were held for 1 min before returning to the initial conditions for 8 min. Column temperature was held constant at 40°C (A: acetonitrile:dichloromethane:NH_3_(aq):HCOOH, 75:25:0.01:0.01, v/v; B: methanol:water:NH_3_(aq):HCOOH, 50:50:0.4:0.4, v/v).

IPLs were identified by exact masses and fragmentation patterns using automated data-dependent fragmentation of base peak ions and compared to commercially available standards and literature data [27, 54, 55]. To quantify changes in the relative abundances of IPLs, peak areas were corrected for their response factors using commercially available standards for phosphatidylglycerol diC16:0 diacylglycerol (16:0 PG), phosphatidylethanolamine diC16:0 diacylglycerol (16:0 PE), C18:1 cardiolipin (18:1 cardiolipin) and monogalactosyl diacylglycerol with multiple fatty acid combinations (MGDG; Avanti Polar Lipids, Inc. USA). Commercial standards for ornithine lipids (OL), *N*-acyl-PE, *N*-acetyl-PE and glycuronic acid diacylglycerol (GADG) standards were not available. Thus, we used the observed range of response factor for tested IPL standards, including diC16:0 phosphatidylcholine (16:0 PC) and digalactosyl diacylglycerol with multiple fatty acid combinations (DGDG; Avanti Polar Lipids, Inc. USA) to assume that apolar compounds that do not ionize very well were underestimated at most eight times relative to PE and that polar compounds that ionize well were overestimated at most three times. The concentrations of injected lipid extracts were adjusted according to the linear range of the instrument, which was three orders of magnitude, from 0.1 to 10 ng on column, for the commercially available standards. Because the concentrations of individual compounds varied strongly within a given sample, formation of dimers caused by high concentrations of analyzed lipids could not always be avoided. Therefore, we considered the peak areas of dimers in the quantification of lipids. S5 and S6 Tables present raw data from lipid analyses and lipid quantification using response factors.

### Epifluorescence, scanning and electron microscopy

Cells analyzed by epifluorescence microscopy and transmission electron microscopy (TEM) grew at 27°C and were harvested during vegetative growth. Cells examined by epifluorescence microscopy were fixed by 2.5% glutaraldehyde in ddH_2_O, stained by Sybr Green stain for nucleic acids (ThermoFischer Scientific, catalog number S7563), imaged using a Zeiss Axio M1 fluorescence microscope (Carl Zeiss Microscopy, LLC), counted and measured using Axiovision Imaging Software (Carl Zeiss Microscopy, LLC). S7 Table presents data used to plot growth curves for wild-type G20 and various mutants and measurements of cell sizes.

Cells harvested for TEM were submerged in fixative comprised of 0.1 M sodium cacodylate, 0.05% CaCl_2_ and 2.5% glutaraldehyde at pH 7.4, and stored at 4°C for at least 2 hours. Samples used for scanning electron microscopy (SEM) were washed three times in the buffer containing 0.1 M sodium cacodylate, 0.05% CaCl_2_ and 0.2M sucrose at pH 7.4, and three times with ddH_2_O, dehydrated in an ethanol series: 30%, 50%, 70%, 80%, 90%, 2x10min 100%; 20 min for each step, filtered through 0.2 μm pore-size polycarbonate filter, coated with a 10 nm-thick Au-Pd coat, and imaged using a field emission Zeiss Supra 55VP SEM with Energy dispersive X-ray spectrometer (EDS) at 10 kV at the Center for Nanoscale Science, Harvard University. Cells analyzed by transmission electron microscopy (TEM) were postfixed with a 1:1 mixture of 2% osmium tetroxide (OsO_4_) and 3% potassium ferrocyanide and washed with ddH_2_O three times. Postfixed and washed samples were incubated in 1% aqueous uranyl acetate for one hour in the dark, washed again with ddH_2_O three times and dehydrated in the following ethanol series: 50%, 70%, 90%, 2x10min 100%; 20 min for each step. After the dehydration, the samples were submerged in propylene oxide for 1 hr and infiltrated by a 1:1 mixture of propylene oxide and Low Viscosity Embedding Media Spurr’s Kit (Electron Microscopy Sciences Catalog number 14300) by overnight shaking. The following day, the cells were embedded in 100% Low Viscosity Spurr’s resin, dried in the oven at 60°C for 48 hours, sectioned and imaged by FEI Technai Transmission Electron Microscope at the W. M. Keck Microscopy Facility at the Whitehead Institute at MIT. The widths of periplasmic spaces and the dimensions of other ultrastructural features were measured in TEM images using the tools in Adobe Illustrator.

The composition of intracellular granules in G20 cells was analyzed by epifluorescence microscopy (Axioplan M2, Carl Zeiss, Inc.) and energy dispersive X-ray spectroscopy (EDS) at the Harvard Center for Nanoscience. To test for the presence of polyphosphate, the cells were stained by 5 μg/ml of DAPI (4,6-diamidino-2-phenylindole, Sigma-Aldrich), incubated for 10 minutes in the dark and imaged with the excitation at 365/10 nm and 474/28 nm and emission at 525/50 nm. The peak of DAPI emission spectrum shifts from 475 nm to 525 nm when DAPI binds to polyphosphate [56] and this method is used to visualize polyphosphate granules in environmental microbes [57]. EDS confirmed the presence of phosphorus in cells grown in MOLS4 containing 200 μM initial phosphate.

To test for the presence of starch, the cells were stained by a 2%KI, 1% I_2_ (w/v) solution, incubated for 10 minutes in the dark and visualized (474/28 nm excitation, 525/50 nm emission) [58]. This method did not reveal the presence of starch or glycogen in G20.

To test for the presence of polyhydroxyalkanoate bodies, unfixed G20 cells were stained by Nile Red (Sigma Aldrich) using a protocol adapted from Rattanapoltee and Kaewkannetra [59]. Briefly, 1-ml aliquots of vegetatively growing cultures were centrifuged for 1 min at 14,000 rpm, resuspended in 100 μl of 25% DMSO, microwaved for 1 min, stained by the addition of 1 mg/ml stock solution of Nile Red stain in DMSO to a final concentration of 10 μg/ml, microwaved for 1 min, incubated in the dark for 10 minutes and centrifuged for 1 min at 14,000 rpm to remove the supernatant. The pellet was resuspended in ~25 μl of the remaining liquid, a drop of this suspension was placed onto the glass slide, and the red fluorescence was visualized using 474/28 nm excitation and 525/50 nm and > 610 nm emission.

## Results

### Growth and cell structure

**Fig 1:**
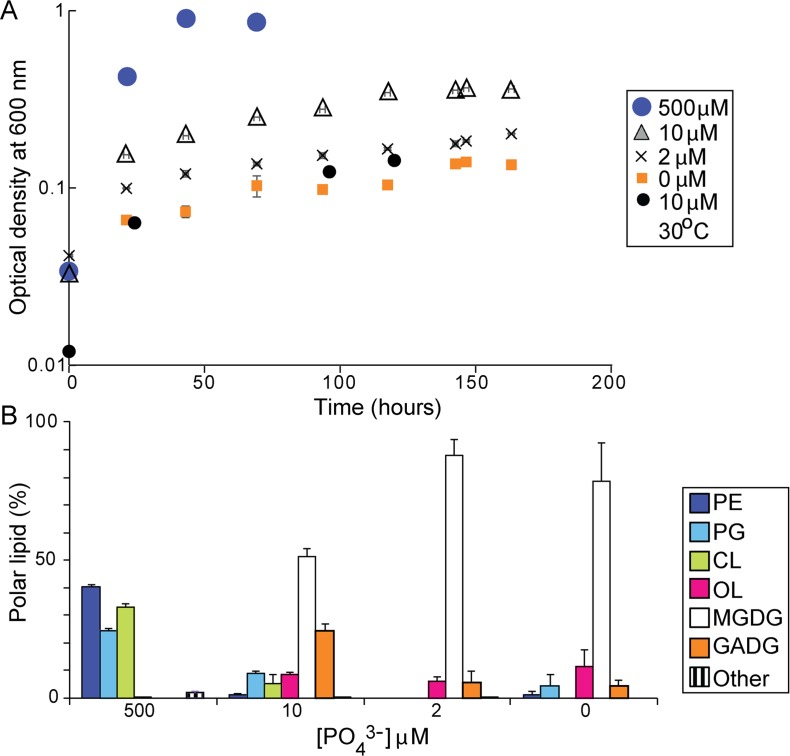
Growth and lipid composition of G20 in media with different initial concentrations of phosphate. (A) Growth at 37°C in duplicate cultures with different initial concentrations of phosphate in the medium and growth at 30°C with 10 μM initial phosphate. (B) The composition of polar lipids in duplicate cultures of G20 grown at 37°C with 500, 10, 2 and 0 μM initial phosphate. PE: phosphatidylethanolamine, PG: phosphatidylglycerol, CL: cardiolipin, OL: ornithine lipids, MGDG: monoglucosyl diacylglycerol, GADG: glycuronic acid diacylglycerol, Other: includes *N*-acetyl-PE, *N*-acyl-PE and two unidentified lipids with head group losses of 199 Da and 213 Da.

*D*. *alaskensis* G20 had lower final cell densities and slower doubling times in batch cultures when the initial concentrations of phosphate were lower than 10 μM (Fig 1A). The composition of cell membranes changed strikingly as a function of phosphate availability. G20 grown in phosphate-replete cultures (500 μM) had membranes composed primarily of phospholipids that were previously described as major lipids in other Desulfovibrio species [60, 61]: phosphatidylethanolamine (PE), phosphatidylglycerol (PG) and cardiolipin (CL) (Fig 1B; S3 Fig). Two additional phospholipids were detected in trace amounts and were identified as *N*-acyl-PE with varying chain lengths in the *N*-acyl head group (S4 Fig) and *N*-acetyl-PE [55]. Furthermore, we observed trace amounts of two unidentified diacylglycerol lipid series with molecular ions in the mass range from *m/z* 700 to 800 and currently structurally unassigned head group losses of 199 Da eluting at 7 min and 213 Da eluting at 8 min. Ornithine lipids (OL) were present in traces, but glycolipids were not detectable (Fig 1B).

In contrast, the membranes of wild-type G20 contained more than 80% of monoglycosyl diacylglycerol (MGDG), glycuronic acid diacylglycerol (MGADG) and ornithine lipids (OL) in all phosphate-limited conditions (Fig 1B, S5 Fig). Moreover, in cultures grown with 2 μM initial phosphate, PE, PG and CL were below the detection limit and phosphorus-free lipids were the only lipids present. Thus, G20 responded to phosphate limitation by synthesizing membranes that contained abundant glyco- and aminolipids.

**Fig 2:**
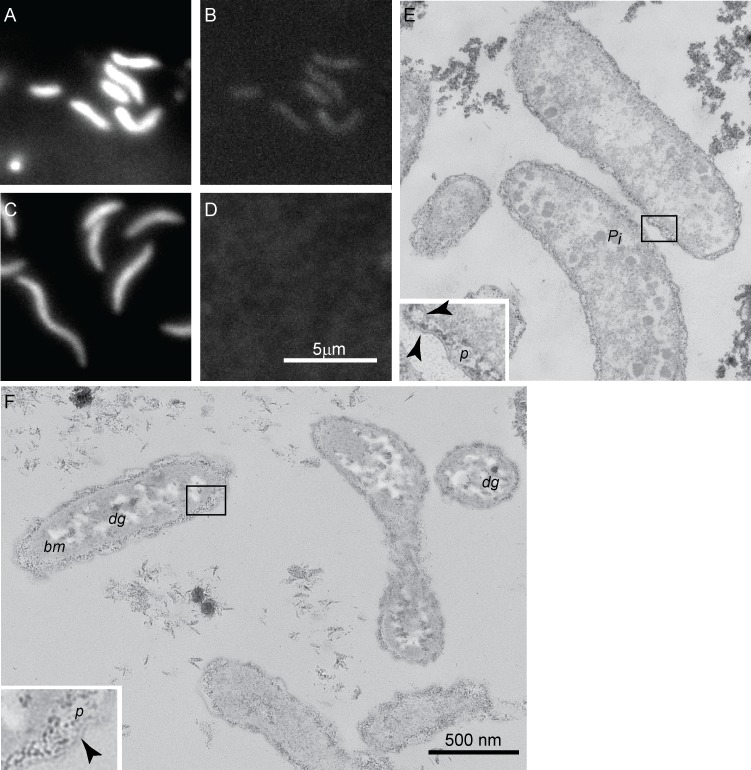
Transmission electron and epifluorescence micrographs of G20 from phosphate-replete and phosphate-limited cultures. (A) Blue fluorescence of DAPI-stained G20 cells not limited by phosphorus. (B) Green fluorescence of DAPI-stained G20 not limited by phosphorus indicates the presence of polyphosphate. (C) Blue fluorescence of elongated, DAPI-stained G20 cells limited by phosphorus. (D) Green fluorescence of DAPI-stained G20 cell limited by phosphate is not detectable due to the absence of polyphosphate granules. Panels (A)-(D) are shown on the same scale. (E) Cells grown at 27°C in MOLS4 with 200 μM initial phosphate. (F) Cells grown at 27°C in MOLS4 with 2 μM initial phosphate. Panels (E) and (F) are shown at the same scale shown in (F). Insets in (E) and (F) show enlarged areas outlined by the black rectangles. Black arrows point to the inner and outer membranes, *p* indicates the periplasm, P*i* marks polyphosphate granules, *bm* labels a representative area filled with bright material, *dg* marks a small dark granule.

Microscopic analyses revealed morphological differences between the cells grown in phosphate-replete and phosphate-limited cultures. The cell lengths were 1.6±0.3 μm (N = 120 cells) when phosphate was plentiful (Fig 2A) and 2.2±0.3 μm (N = 120 cells, p<0.0001) in cultures grown with 2 μM initial phosphate (Fig 2C), but the widths of cells did not change measurably (0.46±0.04 μm vs. 0.47±0.05 μm, N = 120 cells, p<0.14). These changes increased both the surface area and the volume of the curved vibrios in phosphate-limited cultures, but kept a constant ratio of surface area to volume. This increase in biomass per cell contributed to the lower sulfate reduction rates per unit biomass, even though sulfate reduction rates per cell did not change (46.7 fmol sulfide/cell/day produced at 200 μM initial phosphate and 46.2 fmol sulfide/cell/day at 2 μM initial phosphate). Similarly elongated cells and increases in the surface area of some phosphate-limited aerobic bacteria [62] are hypothesized to increase the uptake of phosphorus into the cell [63]. To determine whether the cells contained different amounts of phosphorus, we imaged the green fluorescence of DAPI-stained cells and analyzed their elemental composition by energy dispersive X-ray spectroscopy (Methods). Both approaches detected phosphorus in cells grown with plentiful phosphate (Fig 2B, S6 Fig), and showed that phosphorus was below the detection limit in the cells grown in phosphate-limited medium (Fig 2D, S6 Fig).

To describe ultrastructural changes including those in the composition and distribution of macromolecules in the cell wall, capsule, extracellular material and storage polymers, we imaged the cells by TEM. Cells grown with abundant phosphate had a distinct inner membrane and a crenulated outer membrane, both visible as thin bilayers (Fig 2E). The two membranes enclosed the 17–42 nm wide periplasmic space (average thickness 26 nm, N = 51 points from 3 cells) containing the cell wall composed of dark, granular material. Material with a similar, densely clustered granular appearance was present in the extracellular space. The cytoplasm contained numerous electron-dense granules wider than 40 nm (Fig 2E), likely containing polyphosphate (Fig 2B, S6 Fig). The outer and the inner membranes of cells grown with 2 μM initial phosphate exhibited a lower contrast (Fig 2F) in comparison to the distinct, dark bilayers of cells from phosphate-replete cultures (Fig 2E). The outer membrane of phosphate-limited cells formed larger crenulations and protuberances, expanding the average thickness of the periplasmic space to 36 nm (N = 51 points from 3 cells). The cell wall and exopolymeric substances in the extracellular space were composed of smaller and lighter grains relative to those observed in phosphate-replete cultures. The cytoplasm of phosphate-limited cells contained irregularly shaped regions filled with bright material and occasional small, dark granules (Fig 2F). The bright material resembled storage lipids or glycogen (e.g., [64, 65]) and could also be seen in the TE micrographs of phosphate-limited, nalidixic acid-sensitive G100A [66], the parental strain of G20. To test for the presence of starch granules or neutral lipids, respectively, we stained the cells with KI/I_2_ and Nile red, respectively, but did not observe any fluorescence in KI/I_2_ stained cells or patterns consistent with these storage polymers in cells stained by Nile red (S6 Fig). The disappearance of phosphorus-rich and the synthesis of carbon-rich storage granules pointed to a changed balance between the metabolisms of carbon and phosphorus and was consistent with the reported formation of glycogen by phosphate-limited *Corynebacterium* [67], the formation of PHA in a marine *Pseudovibrio* [62] and the increased C:P ratio in some aerobic environmental bacteria [68].

### Genes important for fitness during phosphate-limited growth and the survival of phosphorus starvation

To better characterize the system-wide response of G20 to phosphate limitation and identify genes that underpin some of the observed compositional and ultrastructural changes, we measured the fitness of a previously described collection of G20 mutants with uniquely tagged and mapped transposon insertions [36]. Because these measurements score fitness as the logarithmic changes in the relative abundances of individual mutants during competitive growth experiments, at least four population doublings are typically required to detect the changed abundances. These conditions cannot be easily met in phosphate-limited cultures of G20 due to low cell densities (Fig 1A), so we developed two different experimental approaches. The first one increased the number of doublings by two sequential transfers in MOLS4 with 10 μM initial phosphate (see Methods) and measured fitness during vegetative growth. The second one probed the survival at three time points during phosphate starvation induced by the depletion of phosphorus in phosphate-limited media (see Methods). This assay measured the relative abilities of mutants to survive starvation and resume growth in nutrient-rich condition. Fitness scores of all genes were determined relative to the scores of the same genes in starting mutant pools grown in MOLS4 with yeast extract (see Methods).

S1 Table presents the fitness scores of all 2338 genes at all analyzed time points. The fitness scores of most genes (2096/2338) exhibited small standard deviations across all conditions (< 0.3) and were therefore excluded from further analyses (see Methods). Where negative, the fitness scores of a gene reflect fitness defects of the corresponding transposon mutants, where positive, they show benefits due to the lack of a gene. To identify genes important for the fitness of G20 in phosphate-limited conditions, we applied numerical criteria described in the Methods to the fitness scores of the remaining genes. Briefly, these criteria searched for genes with fitness defects or benefits that were 1.93-fold larger during phosphate-limited growth than the starting condition and/or genes with fitness defects or benefits that were 1.93-fold larger during phosphate starvation than at the same point during general starvation. The number of genes important for fitness in stationary phase nearly doubled after 15 days (165 genes, 7.1%, S2 Table) and the patterns of their fitness scores were less similar to other data sets (Pearson correlation coefficients lower than 0.75, S3 Table). Hence, we excluded this time point from the analyses shown below.

**Fig 3:**
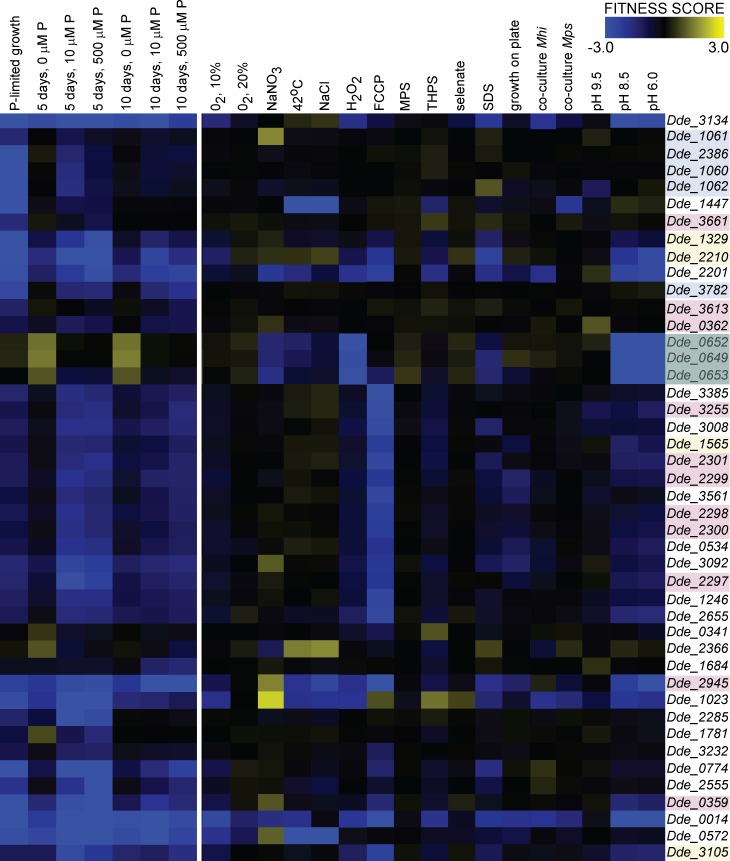
Comparison of fitness scores of genes important during phosphorus-limited growth and phosphate starvation and seventeen other previously tested stresses or growth conditions. Genes with the potential to inform about responses specific to phosphate-limited conditions are listed along the y-axis on the right. Experimental conditions are labeled on top, along the x-axis. The color bar in the top right corner shows colors assigned to the numerical values of fitness scores: negative scores representing fitness defects are blue, positive scores representing fitness benefits are yellow, and fitness-neutral scores are black. The first seven columns starting from the left show scores measured and reported in the current study, the adjacent seventeen columns show scores measured by previous studies and stored in the microbesonline.org database. The names of genes with predicted direct roles in phosphorus homeostasis are labeled by light blue-colored boxes. The names of genes with predicted or confirmed direct roles in the biosynthesis of the cell envelope are labeled by rose-colored boxes. The names of genes with predicted roles in transport are labeled by yellow-colored boxes, those encoding the Hmc complex are labeled by green-colored boxes.

In total, 91 genes were important during phosphate-limited growth and phosphate starvation after 0, 5 and 10 days (Table 1). Based on the similarity of patterns of fitness scores across these experimental conditions, these genes formed nine clusters with Pearson correlation coefficients > 0.62 for the normalized fitness scores within each cluster (Table 1, Fig 3, Methods). Clusters I-IV contained 44 genes with stronger fitness phenotypes during phosphate limitation, phosphate starvation or both. Thus, these genes had a potential to inform about responses specific to the low availability of phosphorus. On the other hand, Clusters V-IX contained 47 genes with large fitness defects (negative scores) in phosphate-replete control cultures or large fitness benefits (positive scores) during phosphate-limited growth. As such, these genes were more important for fitness under conditions of high rates of growth and biosynthesis, i.e., when phosphate was not limiting.

Next, we asked whether the 44 genes with stronger fitness defects in phosphate-limited conditions may also be important for adaptations to other growth conditions or stresses. To do so, we compared their fitness scores measured in our and previous experiments (see Methods). Some of the genes identified in our study also contributed to fitness to the following, previously tested conditions: survival in the presence of 20% and 10% oxygen in the headspace, 150 mM NaNO_3_, incubation at 42°C, 800 mM NaCl, daily addition of 200 μM H_2_O_2_, 100 μg/ml FCCP, 1 mM monofluorophosphate, 0.1 mM THPS, 0.01 mM selenate, sodium dodecyl sulfate (SDS), growth on lactate/sulfate plates, pyruvate fermentation in co-cultures with *Methanospirillum hungatei* (Mh) and *Methanococcus maripaludis* (Mps), respectively, and at pH 9.5, 8.5 and 6 (Fig 3). The following sections describe genes and gene products important during phosphate-limited growth and starvation, discuss the contributions of the same genes to fitness under other conditions and test the roles of genes with predicted functions in the biosynthesis of phosphorus-free lipids.

#### Uptake and metabolism of phosphorus

Five genes important for growth and survival at low phosphate concentrations had direct predicted roles in the uptake of phosphate and regulation of phosphorus metabolism (Table 1). Dde_1060, Dde_1061, Dde_1062 and Dde_2386 are components of the high-affinity phosphate transport system (Pst), whereas Dde_3782 is a homolog of PhoR, a known regulator of the Pho regulon in other bacteria [69]. All five genes had highly correlated fitness scores in our experiments (Pearson correlation coefficient > 0.93) and large defects both during phosphate-limited growth and phosphate starvation (Table 1). Their defects were further exacerbated after 15 days of phosphate starvation (S2 Table). Comparisons with other experimental conditions revealed that these five genes had the largest fitness deficits in phosphate-limited cultures, but were important for growth and survival in very few other conditions (Fig 3). The contribution of PstB, a component of the high-affinity phosphate transporter, is in agreement with the high levels of *pstB* transcripts in phosphate-limited cultures of *G*. *sulfureducens* and *S*. *meliloti* [34, 70]. Other genes with predicted roles in phosphorus homeostasis, such as a phosphate transport regulator Dde_3780, were not important under our experimental conditions or their fitness deficits could not be measured due to the absence of relevant mutants from the mutant pools.

Our fitness assays suggest a complex and temporally variable contribution of PhoU, the negative regulator of the Pho regulon and a global regulator in other bacteria. Phosphate limitation induces the transcription of *phoU* in *G*. *sulfureducens*, and a strain of *S*. *meliloti* [34, 70], and the transcription of *phoU* may be similarly induced in G20. However, our experiments only measured a fitness phenotype in G20 mutants in the *phoU* homolog (Dde_2835) after 15 days of phosphate starvation (S1 and S2 Tables). At this time point, mutants lacking *phoU* (Dde_2835) had enhanced fitness in the two cultures starved for phosphate (fitness score values 8 and 3.5, respectively) relative to the corresponding control culture. This large increase in fitness with the increasing age of the stationary cultures may stem from a metabolically hyperactive status and a reduced frequency of metabolically quiescent bacteria, as reported for *phoU* mutants of *E*.*coli* in stationary phase [71].

G20 likely scavenges phosphorus from nucleic acids, likely from dead cells, during phosphate-limited vegetative growth, as suggested by fitness deficits of a ribonuclease (Dde_1781), a putative transposase (Dde_0534) and Dde_4011, an excinuclease. Additional proteins involved in DNA repair, recombination and RNA processing were important only 15 days after the onset of stationary phase: Dde_0534, a putative transposase, and Dde_0173, a ribonuclease. Strong induction of nuclease-coding genes is also reported in phosphate-limited *B*. *licheniformis* [72].

#### Metabolism and biosynthesis

Various phosphate-limited or starved bacteria have lower abundances of gene transcripts encoding ribosomal proteins or proteins with functions in the synthesis of amino acids, proteins, nucleotides and coenzymes [34, 70, 72]. Mutants in essential genes encoding ribosomal RNA and proteins are absent from the mutant library of G20 and their fitness could not be measured. In spite of this absence, the lower growth rates in phosphate-limited cultures of G20 (Fig 1) imply a much lower content of phosphorus-rich ribosomes and a much reduced cellular requirement for phosphorus during phosphate limitation [19]. This likely explains the lesser importance of most genes with products that had predicted roles in the metabolism and biosynthesis of proteins, amino acids, selenoaminoacids, nucleotides and nucleobases, lipids, quinones, polysaccharides, vitamins and co-factors, the regulation of nitrogen metabolism and glycolysis (Table 1, S7 Fig).

The stronger fitness defect of 1,4 alpha amylase, Dde_2285 during phosphate limitation was an exception to this general trend (Table 1), and is likely involved in the synthesis of abundant bright intracellular granules consistent with glycogen in phosphate-limited cultures (Fig 2).

#### Transport

The fitness of G20 during phosphate limitation and starvation depended on the periplasmic component of ABC-type Mn^2+^/Zn^2+^ transporter (Dde_2210) (Table 1). Based on the large fitness defects of Dde_2210 in the presence of O_2_, the uncoupler FCCP and SDS (Fig 3), lignin, during growth on thiosulfate and in media that lack a reductant, we hypothesize that the transport of Zn^2+^ or Mn^2+^ across the cell membrane contributes to the maintenance of intracellular redox potential and ion gradients during phosphate limitation.

A citrate-dependent iron transporter, Dde_3105, was important during phosphate limitation and starvation (Table 1), but in few other previously tested conditions (Fig 3). The metabolism of phosphorus interacts with the metabolism of iron in some soil bacteria and pathogens [70, 73, 74]. The transcripts of an outer membrane metal efflux protein also increase in phosphate-limited *G*. *sulfurreducens* [34]. Two different ABC-type transporters of dipeptides (Dde_1329, Dde_1565, Table 1) were also important during phosphate-limited growth and starvation. In contrast, phosphate limitation represses the synthesis of two periplasmic peptide permeases in *E*. *coli* [75]. Given that the topological properties and stabilization of LacZ, some amino acid transporters, ion channels, aquaporins and other membrane proteins depend on the presence of specific lipids such as PE, CL or PG [76, 77], changed stabilities and impaired functions of various transporters, or even alternative enzymes can be expected in the phospholipid-poor membranes of G20.

#### General stress response and response regulators

Microbial response to phosphate limitation is thought to activate complex regulatory networks, with diverse downstream effects and cross-talk between stresses [69, 78]. Dde_3092 encodes a heat shock protein and was more important in phosphate-starved cultures after ten days than in the corresponding control culture (Table 1). Phosphate starvation was associated with the greatest fitness defect of Dde_1023, a homolog of DnaK with a weak ATPase activity and function in protein folding and response to heat shock [79] (Table 1, Fig 3). Other genes with predicted regulatory functions, including Dde_1569, a number of genes regulated by the Sigma-54-dependent family of positive activators (Dde_3712, Dde_3713, NtrB Dde_3715 and Dde_3711) and Dde_1260 and Dde_1261, an operon containing a Sigma-54-dependent DNA-binding response regulator and a transcriptional regulator, were more important during general starvation relative to phosphate starvation (Table 1, S7 Fig). The smaller defects of these genes in phosphate-limited cultures are consistent with the slower growth and biosynthetic rates and activity during phosphate starvation.

#### Electron transfer

Hmc, a transmembrane electron transfer complex, had fitness benefits during phosphate-replete stationary phase and was neutral in phosphate-starved cultures (Table 1, Fig 3). Hmc is thought to transport electrons from the cytoplasm to the periplasm and is important for survival in the presence of O_2_ stress, FCCP and for growth on plates (Fig 3). Fumarate reductase (Dde_1256, Dde_1258) and fumarate hydratase (Dde_1254) had fitness benefits during phosphate-limited vegetative growth (Table 1) and were similarly or more mildly detrimental under other conditions when fumarate and malate are not used as the electron donors [42, 50] (S7 Fig). We tentatively attribute these observations to the impact of phosphate limitation on the maintenance of the membrane redox potential.

#### Composition and integrity of the cell envelope during phosphate limitation

Eleven genes with predicted roles in the biosynthesis of the cell envelope and extracellular material, and the maintenance of membrane integrity were important during phosphate limitation or starvation (Table 1, Fig 3). Genes with fitness defects included Dde_0362, a sugar transferase, with an expected role in the biosynthesis of lipopolysaccharide, Dde_0359, a sugar O-acyltransferase similar to NeuD, a protein contributing to the capsular synthesis in *E*. *coli* [80] and Dde_2945, a phosphomannomutase/phosphoglucomutase similar to those with roles in the synthesis of lipopolysaccharide [81]. In contrast, three genes with predicted functions in the biosynthesis of the lipopolysaccharide, Dde_0480, Dde_1806 and Dde_1028 (homolog of AsmA) had fitness defects only during stationary phase in phosphate-replete cultures (Table 1).

Fourteen genes had very similar fitness patterns in our seven experiments (Pearson correlation coefficient > 0.87, p < 0.0001). They had strong fitness defects in the corresponding mutants during phosphate-limited growth or starvation or in the presence of SDS (Fig 3). Among these were Dde_3255, a UDP-n-acetylglucosamine 2-epimerase responsible for the synthesis of the capsular polysaccharides [80] and an operon containing Dde_2297, Dde_2298, Dde_2299, Dde_2300 and Dde_2301. Dde_2298, Dde_2299 and Dde_2301 are the respective homologs of MlaD, MlaF and MlaA, proteins from a complex responsible for the integrity of outer membrane, transport of phospholipids to the inner membrane and the maintenance of membrane asymmetry [82]. *E*. *coli* mutants lacking genes from this operon are more susceptible to lysis in the presence of SDS [82] and the same likely applies to G20 (Fig 3). Based on the observed fitness patterns and changes in the composition of membrane lipids during phosphate limitation (Fig 1B), the *mla* pathway is involved in the maintenance of the outer membrane and lipid trafficking even when phospholipids are almost entirely replaced by glycolipids and ornithine lipids (Fig 1B).

#### Biosynthesis and functions of phosphate-lacking lipids in G20

**Fig 4:**
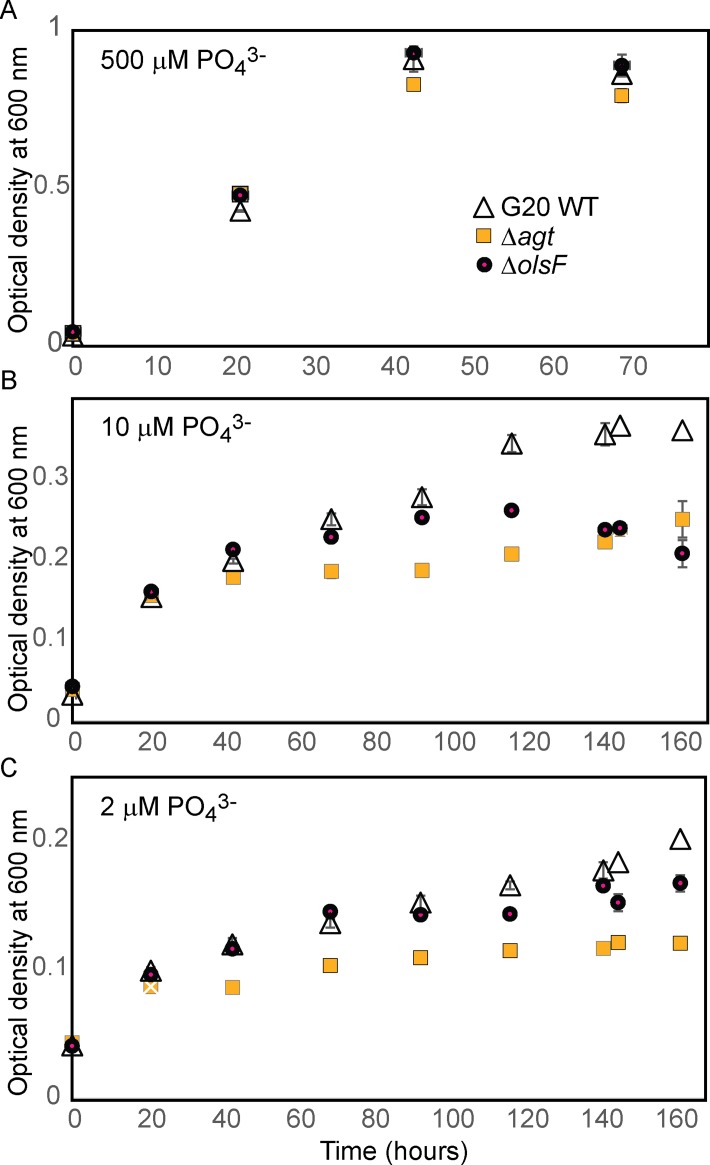
Growth of wild-type G20 and mutants unable to synthesize ornithine lipids, Dde_3661 (Δ*olsF*), or glycolipids,Dde_3613 (Δ*agt*). (A) Growth curves in the presence of 500 μM initial phosphate, when growth is not limited by phosphate. (B) Growth curves in the presence of 10 μM initial phosphate, when growth is limited by phosphate. (C) Growth curves in the presence of 2 μM initial phosphate, when growth is limited by phosphate. Legend in panel (A) applies to all panels.

Mutants in Dde_3613 (*agt*) and Dde_3661 (*olsF*) had fitness defects primarily during vegetative growth in phosphate-limited cultures (Table 1, Fig 4). The closest homologs of Dde_3613 and Dde_3661 are proteins involved in the synthesis of phosphorus-free membrane lipids. Dde_3613 is homologous to Agt, a glycosyltransferase that can synthesize both MGDG and MGADG during phosphate-limited growth of *Agrobacterium tumefaciens* [83]. Dde_3661 is homologous to OlsF, protein that is responsible for the synthesis of ornithine lipids in phosphate-limited *Serratia proteamaculans* [84]. These mutants grew equally fast and attained the same final cell densities as the wild type when the initial concentration of phosphate was 500 μM (Fig 4A). The fitness defects became obvious in cultures containing 10 or 2 μM initial phosphate, where the two mutants grew more slowly and to lower final cell densities than the wild type (Fig 4B and 4C). Of the two mutants, Dde_3613 grew more slowly and had lower final cell densities than Dde_3661, particularly at the lowest initial concentration of phosphate (2 μM, Fig 4C).

**Fig 5:**
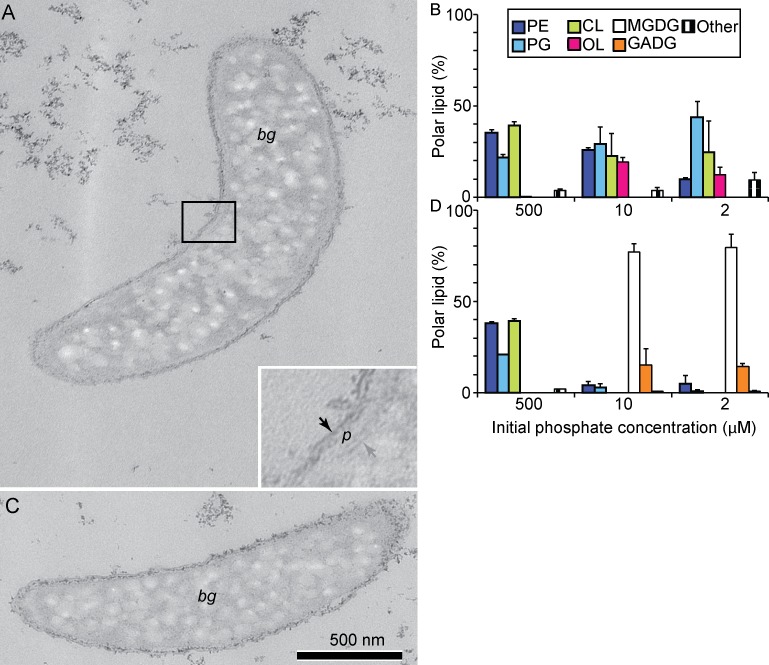
The ultrastructure and composition of polar lipids of Dde_3613 (Δ*agt*) and Dde_3661 (Δ*olsF*). (A) Representative TEM of the Dde_3613 mutant. *Bm* marks a representative area filled with bright material. Black arrows point to the inner and outer membranes, *p* indicates the periplasm. (B) Polar lipid composition of the Dde_3613 mutant grown at different initial phosphate concentrations. (C) Representative TEM of the Dde_3661 mutant. *Bm* marks a representative area filled with bright material. (D) Polar lipid composition of the Dde_3661 mutant grown at different initial phosphate concentrations. (A) and (C) are shown on the same scale shown by the scale bar in panel (C). Abbreviations for the polar head groups: PE is phosphatidylethanolamine, PG is phosphatidylglycerol, CL is cardiolipin, OL is ornithine lipids, MGDG is monoglycosyl diacylglycerol, GADG is glycuronic acid diacylglycerol. Other lipids include N-acyl-PE and N-acetyl-PE and two unidentified lipids with head group losses of 199 Da and 213 Da.

To verify the predicted functions we analyzed the polar membrane lipids of mutants in Dde_3613 and Dde_3661 (Fig 5). When phosphate was abundant, the membranes of both mutants and the wild type had the same composition. However, as expected from the homology between Dde_3613 and Agt, a phosphate-limited Dde_3613 mutant lacked any glycolipids, but contained phospholipids and ornithine lipids (Fig 5B). Ornithine lipids increased from <1% to nearly 20%, and their abundances were comparable to those in phosphate-limited wild-type G20. However, these aminolipids were not able to compensate for the phosphorus demand during membrane lipid biosynthesis at low P concentrations. Instead, the relative amounts of the anionic phospholipids PG, *N*-acyl-PE and *N*-acetyl-PE (shown as “Other” in Fig 5D) increased during phosphate limitation from 25% to over 50%, indicating the importance of these anionic lipids during stress response when neutral glycolipids are lacking (Fig 5D). As expected, ornithine lipids were absent from a phosphate-limited Dde_3661 mutant, which had more than 90% of glycolipids, a higher percentage of phospholipids than the wild type grown under the same conditions and lower cell densities at 2 μM initial phosphate (Figs 2, 4C and 5D).

To test for the presence of ultrastructural differences, we compared the TEM images of phosphate-limited mutants in Dde_3613, Dde_3661 and the wild type (Figs 2 and 5). Only one, smooth membrane bilayer was readily distinguishable on the outer surfaces of both mutants. This membrane enclosed a periplasmic space that exhibited poor contrast with respect to the inner membrane and the cytoplasm (Fig 5A and 5C). Numerous bright granules filled the cytoplasm. The dark, grainy material was present on both sides of the outer membrane (Fig 5) instead of being restricted to the periplasmic space (Fig 2), indicating changes in the composition and distribution of polysaccharides and other macromolecules forming the cell wall, the LPS and the capsule.

OlsF and ornithine lipids in sulfate reducing bacteria are likely to have functions under a broader range of conditions than Agt and glycolipids. For example, eight marine *Desulfovibrio* sp. grown under conditions that are not phosphate-limited have an overall much higher content of ornithine lipids that also depends on the growth temperature [61]. Fitness assays of G20 did not identify Dde_3661 (Δ*olsF*) as more important at 42°C than 30°C, but mutants in this gene have mildly enhanced fitness in the presence of 1 mM Tetrakis(hydoxymethyl)phosphonium sulfate (THPS) and 1 mM monofluorophosphate (MFP, FPO_3_^2-^) (fitness score values 0.69 and 0.5, respectively). THPS is commonly used in the oil industry to prevent the growth of sulfate reducing bacteria [3] and its mode of action involves the release of reactive aldehydes that cross-link cell envelopes and proteins. MFP is a selective inhibitor of the sulfate reduction pathway and also releases cytoplasmic fluoride ion, inhibiting enzymes that rely on hydroxide ion for catalysis (e.g. enolase) [51]. Therefore, we hypothesize that treatments of sulfate reducing microbes with MFP or THPS may be more efficient during phosphate limitation.

A Dde_3613 mutant grew poorly in phosphate-limited media (Fig 4B and 4C). This is consistent both with the roles of MGDG and GADG as the major substitutes for phospholipids in wild-type G20 and the larger proportion of phospholipids in this mutant (Fig 5). Because Dde_3613 (Agt) was only important for fitness during phosphate limitation (Fig 3) and G20 did not contain even trace amounts of glycolipids during growth with 500 μM initial phosphate (Fig 1B), glycolipids appear to be produced specifically in response to phosphate limitation. To the best of our knowledge, glycolipids were not detected by previous studies of ∂-Proteobacteria, although the genomes of more than 50 other species and strains contain *agt* homologs (BLAST threshold e-value < e^-100^). Based on our data, we predict that these microbes will synthesize glycolipids only when limited by phosphate.

## Discussion

Studies of phosphate limitation have greatly benefited from the genetic tractability of *E*. *coli* and *B*. *subtilis*, but the responses of these organisms may differ greatly from those of environmental microbes adapted to growth at low phosphate concentrations [19, 68]. Various marine Alphaproteobacteria and Flavobacteria remodel their lipids during phosphorus starvation, but appear to do so less completely, retaining 20% or more of phospholipids [18, 85]. The near absence of phospholipids is reported in phosphate-limited continuous cultures of Gammaproteobacteria *Pseudomonas fluorescens* NCMB 179 and *P*. *diminuta* [16, 17], as well as in early stationary-phase cultures of phosphate-limited freshwater Actinobacteria, Alpha- and Gammaproteobacteria [19]. Although these phylogenetically and physiologically diverse bacteria exhibit a similar trend toward phosphorus-lacking lipids, the synthesis of MGDG as the major glycolipid appears to be a distinct feature of phosphorus-limited G20. Similar prevalence of MGDG was previously reported in environmental samples [20, 21], but not in culture experiments.

The presence of neutral MGDG as the main membrane lipid in phosphate-limited G20 and the frequency of very similar *agt* homologs in numerous ∂-Proteobacteria suggest that: 1. These organisms contribute to the levels of MGDG in the suboxic and the anoxic zones of the Black Sea and the Eastern Northern Tropical Pacific [20, 21] and 2. Microbes at these sites experience phosphorus limitation. Other possible sources of MGDG in these environments are anaerobic or microaerophilic microbes with *ag*t homologs, e.g., Epsilonproteobacteria (*Arcobacter*, *Sulfurovum* and *Thiovulum sp*.) and some Planctomycetes, but it remains to be determined whether they accumulate MGDG or other lipids under phosphate-limiting conditions. Further confirmation of widespread phosphorus limitation in these environments may also come from transcriptomic studies, if transcripts of *agt* and components of the Pho regulon in environmental samples occur in the same samples.

Our study shows the nearly complete replacement of phosphorus containing lipids by lipids devoid of that element in a sulfate reducing bacterium under laboratory conditions. Furthermore, our data demonstrate additional changes in the composition of the cell envelope (Fig 1, Table 1) during phosphate-limited growth of G20 and identify various genes that are important for fitness both during phosphate limitation or the survival of phosphorus starvation, as well as in the presence of stresses induced by environmental factors and man-made inhibitors (Fig 3). These observations suggest that the availability of phosphorus may modify the microbial response to various environmental and anthropogenic stressors. System-wide assays and mutant libraries provide valuable tools by which to test this prediction.

## Conclusions

Slower growth, nearly complete replacement of phospholipids by glycolipids and ornithine lipids, synthesis of carbon-rich intracellular granules, an increase in the cell length and changed appearances of the outer membranes, peptidoglycan and exopolymeric substances accompany phosphate-limited growth of the sulfate reducing bacterium *Desulfovibrio alaskensis* G20 at < 20 μM PO_4_^3-^. Fitness analyses with a library of transposon mutants identify 91 genes important for the adaptation to phosphate-limited growth and starvation including genes involved in high-affinity phosphate uptake, transport, stress response and those that control the composition and structure of the cell envelope. Lipid analyses of mutants lacking Dde_3613 and Dde_3661, respectively, confirm the roles of these genes in the synthesis of glycolipids and ornithine lipids, respectively. Fitness assays predict impacts of phosphate limitation on the microbial responses to other environmental stresses, biocides, inhibitors and organisms. The identification of MGDG as the main phosphorus-free lipid in growing cultures of phosphate-limited G20 supports the interpretation of abundant MGDG in some anaerobic marine environments as a stoichiometric signal of phosphate limitation. This signal is likely produced by heterotrophic and/or chemolithotrophic microbes that live at micromolar concentrations of phosphate.

## Supporting Information

S1 Fig Fitness patterns of genes important during phosphate-limited growth and for the survival of and recovery after phosphate starvation. Hierarchical clustering analysis identified nine clusters with distances smaller than 0.5 using the Pearson correlation coefficient as a distance metric and average linkage clustering. “Veg” denotes vegetatively growing pool cultures and “stat” denotes cultures in stationary phase after 5 or 10 days of starvation at 0, 10 or 500 μM initial phosphate in the medium.
(JPG)

S2 Fig Growth of wild-type G20 and four mutants carrying transposons in genes important during phosphate limited growth: Dde_2285, Dde_1023, Dde_3255 and Dde_1565. All mutants had fitness defects in pure cultures as well as in mutant pool experiments. G20 WT is the wild-type G20.
(JPG)

S3 Fig Representative HPLC-MS chromatograms showing changes in the polar membrane composition of G20. (A) Wild-type G20 in phosphate-replete cultures. (B) Dde_3613 (Δ*agt*) in phosphate-limited culture. (C) Wild-type G20 in phosphate-limited cultures. (D) Dde_3661 (Δ*olsF*) in phosphate-limited culture. The HPLC-MS chromatograms are depicted as density maps. The x-axis shows the retention time, the y-axis shows *m/z*, the relative peak intensity is shown in color. PE: phosphatidylethanolamine, PG: phosphatidylglycerol, CL: cardiolipin, OL: ornithine lipids, MGDG: monoglycosyl diacylglycerol, GADG: glycuronic acid diacylglycerol. Dimers form in the ion source during elevated concentrations of analyzed compounds.
(TIF)

S4 Fig Identification of *N*-acylphosphatidylethanolamine (*N*-acyl-PE) and *N*-acetylphosphatidylethanolamine (*N*-acetyl-PE) by high-resolution accurate-mass quadrupole time of flight mass spectrometry in positive ion mode. (A) Dominant protonated *N*-acyl-PE ions detected during full scan (MS^1^) at 7.5–8 min reflect typical acyl chain heterogeneity. The star (*) denotes complementary ammonium adducts of the protonated molecular ions. (B) High-resolution accurate-mass quadrupole MS^2^ mass spectrum of *N*-acyl-PE ions *m/z* 956.768 and *m/z* 942.768. The chemical formulas represent neutral losses and products after MS^2^ fragmentation. As with other multiple-acylated intact polar lipids, a given molecular mass of *N*-acyl-PE can represent several distinct molecular species. Changes in the acyl chain length can occur both in the diacylglycerol core lipid or the *N*-acyl-PE head group. For instance, at least two species can explain the ion *m/z* 956.768, one with a combined diacylglycerol acyl chain length of C32:1 and *N*-acyl-PE acyl chain length of C17:0 or a combination of C33:1 and C16:0 for diacylglycerol or *N*-acyl-PE acyl chains, respectively. (C_ Dominant protonated *N*-acetyl-PE ions detected during full scan (MS^1^) at 8.5–9.5 min. The star (*) denotes the complementary ammonium adducts to the protonated molecular ions. (D) High-resolution accurate-mass quadrupole MS^2^ mass spectrum of *N*-acetyl-PE ions *m/z* 732.517 and *m/z* 720.517. The neutral loss of 183.03 observed for these ions indicates the loss of the *N*-acetyl-PE head group.
(TIF)

S5 Fig Identification of ornithine lipids (OL) and monoglycosyl diacylglycerol (MGDG) by high-resolution accurate-mass quadrupole time of flight mass spectrometry in positive ion mode. (A) Dominant protonated OL ions detected during full scan (MS^1^) at 12.5–13 min reflect typical acyl chain heterogeneity. (B) High-resolution accurate-mass quadrupole MS^2^ mass spectrum of OL ion *m/z* 639.567, showing major fragments and their chemical formulas. As with other multiple-acylated intact polar lipids, a given molecular mass of OL can represent several distinct molecular species. At least two species can explain the ion *m/z* 639.567, one with an acyl chain length of C16:0 for the ß-OH fatty acid amide-linked to the ornithine headgroup and a C17:0 acyl chain length for the fatty acid esterified to the hydroxyl group and vice versa. (C) Dominant MGDG ions with ammonium adducts detected during full scan (MS^1^) at 4–5 min. (D) High-resolution accurate-mass quadrupole MS^2^ mass spectrum of MGDG ion *m/z* 788.626. The chemical formulas represent neutral losses and products after MS^2^ fragmentation.
(TIF)

S6 Fig Phosphorus content and the staining patterns of G20 stained by Nile red. (A) EDS spectrum of cells grown in the phosphate-replete medium contains a phosphorus peak. Inset: Scanning electron micrograph of analyzed cells on a polycarbonate filter. (B) EDS spectrum of cells grown in phosphate-limited medium lack a detectable phosphorus peak. All samples were coated by Au and Pd. (C) Epifluorescence micrograph of cells stained by Nile red after growth in phosphate replete medium. (D) Epifluorescence micrograph of cells stained by Nile red after growth in phosphate limited medium. The scale shown in panel (D) applies to both panels (C) and (D).
(JPG)

S7 Fig Fitness scores of important genes from Clusters V-IX in our experiments and in the presence of other stresses. The top seven rows show fitness scores measured in our experiments. Subsequent rows show fitness scores of the same genes during the survival in the presence of 20% and 10% oxygen in the headspace, 150 mM NaNO_3_, incubation at 42°C, 800 mM NaCl, the daily addition of 200 μM H_2_O_2_, 100 μg/ml FCCP, 1 mM monofluorophosphate, 0.1 mM THPS, 0.01 mM selenate, sodium dodecyl sulfate (SDS), growth on lactate/sulfate plates, pyruvate fermentation in co-cultures with *Methanospirillum hungatei* (Mh) and *Methanococcus maripaludis* (Mps), respectively, and at pH 9.5, 8.5 and 6.
(JPG)

S1 Table Fitness scores of all 2338 genes at all analyzed time points. (ZIP)

S2 Table Genes important for survival after 15 days of phosphate starvation. Genes are important if: 1. their fitness scores differ by more than 0.95 between the phosphate replete culture in stationary phase and the two phosphate starved cultures; 2. the fitness score during P-limited vegetative growth is > 0.95 or <-0.95, and more than 0.58 units different from the scores in phosphate starved or phosphate replete culture sampled 15 days after the onset of stationary phase. Colors mark clusters of genes with Pearson correlation coefficients >0.65. Genes with italicized functions are also important at previous time points or only during vegetative growth in phosphate-limited MOLS4.
(XLSX)

S3 Table Correlation coefficients of fitness scores of all genes measured in the seven conditions. (DOCX)

S4 Table Compressed Excel file with raw fitness data. (ZIP)

S5 Table Raw data from lipid analyses. (XLSX)

S6 Table Raw data showing the quantification of lipids with response factors. (XLSX)

S7 Table Raw data showing growth curves for G20 and various mutants and cell size measurements. (XLSX)

## References

[1] Rabus R, Hansen TA, Widdel F. Dissimilatory sulfate-and sulfur-reducing prokaryotes The prokaryotes: Springer; 2006 p. 659–768.

[2] Muyzer G, Stams AJ. The ecology and biotechnology of sulphate-reducing bacteria. Nature Reviews Microbiology. 2008;6(6):441–54. 10.1038/nrmicro1892 18461075

[3] Lee M-HP, Caffrey SM, Voordouw JK, Voordouw G. Effects of biocides on gene expression in the sulfate-reducing bacterium *Desulfovibrio vulgaris* Hildenborough. Applied microbiology and biotechnology. 2010;87(3):1109–18. 10.1007/s00253-010-2596-1 20437234

[4] Chhabra S, He Q, Huang K, Gaucher S, Alm E, He Z, et al Global analysis of heat shock response in *Desulfovibrio vulgaris* Hildenborough. Journal of bacteriology. 2006;188(5):1817–28. 10.1128/JB.188.5.1817-1828.2006 16484192 PMC1426554

[5] Mukhopadhyay A, He Z, Alm EJ, Arkin AP, Baidoo EE, Borglin SC, et al Salt stress in *Desulfovibrio vulgaris* Hildenborough: an integrated genomics approach. Journal of bacteriology. 2006;188(11):4068–78. 10.1128/JB.01921-05 16707698 PMC1482918

[6] Mukhopadhyay A, Redding AM, Joachimiak MP, Arkin AP, Borglin SE, Dehal PS, et al Cell-wide responses to low-oxygen exposure in *Desulfovibrio vulgaris* Hildenborough. Journal of bacteriology. 2007;189(16):5996–6010. 10.1128/JB.00368-07 17545284 PMC1952033

[7] He Q, Huang KH, He Z, Alm EJ, Fields MW, Hazen TC, et al Energetic consequences of nitrite stress in *Desulfovibrio vulgaris* Hildenborough, inferred from global transcriptional analysis. Applied and environmental microbiology. 2006;72(6):4370–81. 10.1128/AEM.02609-05 16751553 PMC1489655

[8] Stolyar S, He Q, Joachimiak MP, He Z, Yang ZK, Borglin SE, et al Response of *Desulfovibrio vulgaris* to alkaline stress. Journal of bacteriology. 2007;189(24):8944–52. 10.1128/JB.00284-07 17921288 PMC2168612

[9] Zhang W, Culley DE, Hogan M, Vitiritti L, Brockman FJ. Oxidative stress and heat-shock responses in Desulfovibrio vulgaris by genome-wide transcriptomic analysis. Antonie Van Leeuwenhoek. 2006;90(1):41–55. 10.1007/s10482-006-9059-9 16680520

[10] Sim MS, Ono S, Bosak T. Effects of iron and nitrogen limitation on sulfur isotope fractionation during microbial sulfate reduction. Applied and environmental microbiology. 2012;78(23):8368–76. 10.1128/AEM.01842-12 23001667 PMC3497358

[11] Benitez-Nelson CR. The biogeochemical cycling of phosphorus in marine systems. Earth-Science Reviews. 2000;51(1):109–35.

[12] Wu J, Sunda W, Boyle EA, Karl DM. Phosphate depletion in the western North Atlantic Ocean. Science. 2000;289(5480):759–62. 10926534 10.1126/science.289.5480.759

[13] Benning C, Huang Z-H, Gage DA. Accumulation of a novel glycolipid and a betaine lipid in cells of *Rhodobacter sphaeroides* grown under phosphate limitation. Archives of biochemistry and biophysics. 1995;317(1):103–11. 10.1006/abbi.1995.1141 7872771

[14] Ellwood D, Tempest D. Control of teichoic acid and teichuronic acid biosynthesis in chemostat cultures of *Bacillus subtilis* var. *niger*. Biochemical Journal. 1969;111(1):1–5. 4975313 10.1042/bj1110001 PMC1187486

[15] Van Mooy BA, Fredricks HF, Pedler BE, Dyhrman ST, Karl DM, Koblížek M, et al Phytoplankton in the ocean use non-phosphorus lipids in response to phosphorus scarcity. Nature. 2009;458(7234):69–72. 10.1038/nature07659 19182781

[16] Minnikin D, Abdolrahimzadeh H. The replacement of phosphatidylethanolamine and acidic phospholipids by an ornithine‐amide lipid and a minor phosphorus‐free lipid in *Pseudomonas flourescens* NCMB 129. FEBS letters. 1974;43(3):257–60. 4213476 10.1016/0014-5793(74)80655-1

[17] Minnikin D, Abdolrahimzadeh H, Baddiley J. Replacement of acidic phospholipids by acidic glycolipids in Pseudomonas diminuta. 1974. 10.1038/249268a0 4833243

[18] Sebastián M, Pitta P, González JM, Thingstad TF, Gasol JM. Bacterioplankton groups involved in the uptake of phosphate and dissolved organic phosphorus in a mesocosm experiment with P‐starved Mediterranean waters. Environmental microbiology. 2012;14(9):2334–47. 10.1111/j.1462-2920.2012.02772.x 22564346

[19] Yao M, Elling FJ, Jones C, Nomosatryo S, Long CP, Crowe SA, et al Heterotrophic bacteria from an extremely phosphate‐poor lake have conditionally reduced phosphorus demand and utilize diverse sources of phosphorus. Environmental microbiology. 2015. 10.1111/1462-2920.13063 PMC5872838 26415900

[20] Schubotz F, Wakeham SG, Lipp JS, Fredricks HF, Hinrichs KU. Detection of microbial biomass by intact polar membrane lipid analysis in the water column and surface sediments of the Black Sea. Environmental Microbiology. 2009;11(10):2720–34. 10.1111/j.1462-2920.2009.01999.x 19624710

[21] Braun S, Morono Y, Becker KW, Hinrichs K-U, Kjeldsen KU, Jørgensen BB, et al Cellular content of biomolecules in sub-seafloor microbial communities. Geochimica et Cosmochimica Acta. 2016;188:330–51.

[22] Rossel PE, Elvert M, Ramette A, Boetius A, Hinrichs K-U. Factors controlling the distribution of anaerobic methanotrophic communities in marine environments: evidence from intact polar membrane lipids. Geochimica et Cosmochimica Acta. 2011;75(1):164–84.

[23] Millero FJ. Chemical oceanography Boca Raton: CRC/Taylor and Francis, 2006. 3rd ed.; 2006. 530 p.

[24] Flohr A, Van der Plas A, Emeis K, Mohrholz V, Rixen T. Spatio-temporal patterns of C: N: P ratios in the northern Benguela upwelling system. Biogeosciences. 2014;11(3):885–97.

[25] Ingall E, Jahnke R. Evidence for enhanced phosphorus regeneration from marine sediments overlain by oxygen depleted waters. Geochimica et Cosmochimica Acta. 1994;58(11):2571–5.

[26] Paytan A, McLaughlin K. The oceanic phosphorus cycle. Chemical reviews. 2007;107(2):563–76. 10.1021/cr0503613 17256993

[27] Geiger O, Röhrs V, Weissenmayer B, Finan TM, Thomas‐Oates JE. The regulator gene *phoB* mediates phosphate stress‐controlled synthesis of the membrane lipid diacylglyceryl‐N, N, N‐trimethylhomoserine in *Rhizobium (Sinorhizobium) meliloti*. Molecular microbiology. 1999;32(1):63–73. 10216860 10.1046/j.1365-2958.1999.01325.x

[28] Yuasa J, Nakagawa Y, Kotani Y, Shimohata A, Matsuyama T. *Pseudomonas aeruginosa* under phosphorus-poor culture conditions: Phospholipid-poor bacterial membranes, and susceptibility to antibacterial chemicals, high temperature and low pH. Microbes and environments. 2002;17(2):75–81.

[29] Canfield DE, Stewart FJ, Thamdrup B, De Brabandere L, Dalsgaard T, Delong EF, et al A cryptic sulfur cycle in oxygen-minimum–zone waters off the Chilean coast. Science. 2010;330(6009):1375–8. 10.1126/science.1196889 21071631

[30] Lin X, Wakeham SG, Putnam IF, Astor YM, Scranton MI, Chistoserdov AY, et al Comparison of vertical distributions of prokaryotic assemblages in the anoxic Cariaco Basin and Black Sea by use of fluorescence in situ hybridization. Applied and Environmental Microbiology. 2006;72(4):2679–90. 10.1128/AEM.72.4.2679-2690.2006 16597973 PMC1449015

[31] Madrid VM, Taylor GT, Scranton MI, Chistoserdov AY. Phylogenetic diversity of bacterial and archaeal communities in the anoxic zone of the Cariaco Basin. Applied and environmental microbiology. 2001;67(4):1663–74. 10.1128/AEM.67.4.1663-1674.2001 11282619 PMC92783

[32] Stewart FJ. Dissimilatory sulfur cycling in oxygen minimum zones: an emerging metagenomics perspective. Biochemical Society Transactions. 2011;39(6):1859–63. 10.1042/BST20110708 22103540

[33] Wakeham SG, Amann R, Freeman KH, Hopmans EC, Jørgensen BB, Putnam IF, et al Microbial ecology of the stratified water column of the Black Sea as revealed by a comprehensive biomarker study. Organic Geochemistry. 2007;38(12):2070–97.

[34] N'Guessan AL, Elifantz H, Nevin KP, Mouser PJ, Methé B, Woodard TL, et al Molecular analysis of phosphate limitation in Geobacteraceae during the bioremediation of a uranium-contaminated aquifer. The ISME journal. 2010;4(2):253–66. 10.1038/ismej.2009.115 20010635

[35] Xia Q, Wang T, Hendrickson EL, Lie TJ, Hackett M, Leigh JA. Quantitative proteomics of nutrient limitation in the hydrogenotrophic methanogen *Methanococcus maripaludis*. BMC microbiology. 2009;9(1):1. 19627604 10.1186/1471-2180-9-149 PMC2723118

[36] Kuehl JV, Price MN, Ray J, Wetmore KM, Esquivel Z, Kazakov AE, et al Functional genomics with a comprehensive library of transposon mutants for the sulfate-reducing bacterium *Desulfovibrio alaskensis* G20. MBio. 2014;5(3):e01041–14. 10.1128/mBio.01041-14 24865553 PMC4045070

[37] Zane GM, Yen H-cB, Wall JD. Effect of the deletion of *qmoABC* and the promoter-distal gene encoding a hypothetical protein on sulfate reduction in *Desulfovibrio vulgaris* Hildenborough. Applied and environmental microbiology. 2010;76(16):5500–9. 10.1128/AEM.00691-10 20581180 PMC2918943

[38] Brandis A, Thauer RK. Growth of Desulfovibrio species on hydrogen and sulphate as sole energy source. Microbiology. 1981;126(1):249–52.

[39] Cline JD, Richards FA. Oxygenation of hydrogen sulfide in seawater at constant salinity, temperature and pH. Environmental Science & Technology. 1969;3(9):838–43.

[40] Sim MS, Ono S, Donovan K, Templer SP, Bosak T. Effect of electron donors on the fractionation of sulfur isotopes by a marine *Desulfovibrio* sp. Geochimica et Cosmochimica Acta. 2011;75(15):4244–59.

[41] Sim MS, Bosak T, Ono S. Large sulfur isotope fractionation does not require disproportionation. Science. 2011;333(6038):74–7. 10.1126/science.1205103 21719675

[42] Price MN, Ray J, Wetmore KM, Kuehl JV, Bauer S, Deutschbauer AM, et al The genetic basis of energy conservation in the sulfate-reducing bacterium *Desulfovibrio alaskensis* G20. Frontiers in microbiology. 2014;5:577 10.3389/fmicb.2014.00577 25400629 PMC4215793

[43] Oh J, Fung E, Price MN, Dehal PS, Davis RW, Giaever G, et al A universal TagModule collection for parallel genetic analysis of microorganisms. Nucleic acids research. 2010;38(14):e146–e. 10.1093/nar/gkq419 20494978 PMC2919733

[44] Oh J, Fung E, Schlecht U, Davis RW, Giaever G, Onge RPS, et al Gene annotation and drug target discovery in *Candida albicans* with a tagged transposon mutant collection. PLoS Pathog. 2010;6(10):e1001140 10.1371/journal.ppat.1001140 20949076 PMC2951378

[45] Pierce SE, Davis RW, Nislow C, Giaever G. Genome-wide analysis of barcoded *Saccharomyces cerevisiae* gene-deletion mutants in pooled cultures. Nature protocols. 2007;2(11):2958–74. 10.1038/nprot.2007.427 18007632

[46] Wetmore KM, Price MN, Waters RJ, Lamson JS, He J, Hoover CA, et al Rapid quantification of mutant fitness in diverse bacteria by sequencing randomly bar-coded transposons. MBio. 2015;6(3):e00306–15. 10.1128/mBio.00306-15 25968644 PMC4436071

[47] Saeed A, Bhagabati N, Braisted J, Sturn A, Quackenbush J. TIGR MeV Multiexperiment Viewer. The Institute for Genomic Research 2003.

[48] Eisen MB, Spellman PT, Brown PO, Botstein D. Cluster analysis and display of genome-wide expression patterns. Proceedings of the National Academy of Sciences. 1998;95(25):14863–8. 10.1073/pnas.95.25.14863 PMC24541 9843981

[49] Dehal PS, Joachimiak MP, Price MN, Bates JT, Baumohl JK, Chivian D, et al MicrobesOnline: an integrated portal for comparative and functional genomics. Nucleic acids research. 2010;38(suppl 1):D396–D400. 19906701 10.1093/nar/gkp919 PMC2808868

[50] Meyer B, Kuehl JV, Price MN, Ray J, Deutschbauer AM, Arkin AP, et al The energy‐conserving electron transfer system used by *Desulfovibrio alaskensis* strain G20 during pyruvate fermentation involves reduction of endogenously formed fumarate and cytoplasmic and membrane‐bound complexes, Hdr‐Flox and Rnf. Environmental microbiology. 2014;16(11):3463–86. 10.1111/1462-2920.12405 24447568

[51] Carlson HK, Stoeva MK, Justice NB, Sczesnak A, Mullan MR, Mosqueda LA, et al Monofluorophosphate is a selective inhibitor of respiratory sulfate-reducing microorganisms. Environmental science & technology. 2015;49(6):3727–36. 25698072 10.1021/es505843z

[52] Sturt HF, Summons RE, Smith K, Elvert M, Hinrichs KU. Intact polar membrane lipids in prokaryotes and sediments deciphered by high‐performance liquid chromatography/electrospray ionization multistage mass spectrometry—new biomarkers for biogeochemistry and microbial ecology. Rapid Communications in Mass Spectrometry. 2004;18(6):617–28. 10.1002/rcm.1378 15052572

[53] Wörmer L, Lipp JS, Schröder JM, Hinrichs K-U. Application of two new LC–ESI–MS methods for improved detection of intact polar lipids (IPLs) in environmental samples. Organic Geochemistry. 2013;59:10–21.

[54] Schubotz F, Xie S, Wakeham SG, Hinrichs K-U. Non-phosphorus lipids in the oxygen minimum zone of the eastern tropical North Pacific. submitted.

[55] Mileykovskaya E, Ryan AC, Mo X, Lin C-C, Khalaf KI, Dowhan W, et al Phosphatidic acid and N-acylphosphatidylethanolamine form membrane domains in Escherichia coli mutant lacking cardiolipin and phosphatidylglycerol. Journal of Biological Chemistry. 2009;284(5):2990–3000. 10.1074/jbc.M805189200 19049984 PMC2631977

[56] Aschar-Sobbi R, Abramov AY, Diao C, Kargacin ME, Kargacin GJ, French RJ, et al High sensitivity, quantitative measurements of polyphosphate using a new DAPI-based approach. Journal of fluorescence. 2008;18(5):859–66. 10.1007/s10895-008-0315-4 18210191

[57] Bar-Yosef Y, Sukenik A, Hadas O, Viner-Mozzini Y, Kaplan A. Enslavement in the water body by toxic *Aphanizomenon ovalisporum*, inducing alkaline phosphatase in phytoplanktons. Current biology. 2010;20(17):1557–61. 10.1016/j.cub.2010.07.032 20705465

[58] Ovecka M, Bahaji A, Muñoz FJ, Almagro G, Ezquer I, Baroja-Fernández E, et al A sensitive method for confocal fluorescence microscopic visualization of starch granules in iodine stained samples. Plant signaling & behavior. 2012;7(9):1146–50. 22899048 10.4161/psb.21370 PMC3489648

[59] Rattanapoltee P, Kaewkannetra P. Nile red, an alternative fluorescence method for quantification of neutral lipids in microalgae. World Academy of Science, Engineering and Technology, International Journal of Biological, Biomolecular, Agricultural, Food and Biotechnological Engineering. 2013;7(9):889–93.

[60] Makula R, Finnerty W. Phospholipid composition of Desulfovibrio species. Journal of bacteriology. 1974;120(3):1279–83. 4436257 10.1128/jb.120.3.1279-1283.1974 PMC245912

[61] Seidel M, Rütters H, Rullkötter J, Sass H. Phosphate-free ornithine lipid contents in *Desulfovibrio* spp. respond to growth temperature. Organic geochemistry. 2013;59:133–42.

[62] Romano S, Schulz-Vogt HN, González JM, Bondarev V. Phosphate limitation induces drastic physiological changes, virulence-related gene expression, and secondary metabolite production in Pseudovibrio sp. strain FO-BEG1. Applied and environmental microbiology. 2015;81(10):3518–28. 10.1128/AEM.04167-14 25769826 PMC4407226

[63] Thingstad TF, Øvreås L, Egge JK, Løvdal T, Heldal M. Use of non‐limiting substrates to increase size; a generic strategy to simultaneously optimize uptake and minimize predation in pelagic osmotrophs? Ecology Letters. 2005;8(7):675–82.

[64] Jendrossek D, Selchow O, Hoppert M. Poly (3-hydroxybutyrate) granules at the early stages of formation are localized close to the cytoplasmic membrane in *Caryophanon latum*. Applied and environmental microbiology. 2007;73(2):586–93. 10.1128/AEM.01839-06 17085698 PMC1796971

[65] Alonso-Casajús N, Dauvillée D, Viale AM, Muñoz FJ, Baroja-Fernández E, Morán-Zorzano MT, et al Glycogen phosphorylase, the product of the *glgP* gene, catalyzes glycogen breakdown by removing glucose units from the nonreducing ends in *Escherichia coli*. Journal of bacteriology. 2006;188(14):5266–72. 10.1128/JB.01566-05 16816199 PMC1539952

[66] Weimer PJ, Van Kavelaar MJ, Michel CB, Ng TK. Effect of phosphate on the corrosion of carbon steel and on the composition of corrosion products in two-stage continuous cultures of *Desulfovibrio desulfuricans*. Applied and environmental microbiology. 1988;54(2):386–96. 16347552 10.1128/aem.54.2.386-396.1988 PMC202462

[67] Woo HM, Noack S, Seibold GM, Willbold S, Eikmanns BJ, Bott M. Link between phosphate starvation and glycogen metabolism in *Corynebacterium glutamicum*, revealed by metabolomics. Applied and environmental microbiology. 2010;76(20):6910–9. 10.1128/AEM.01375-10 20802079 PMC2953031

[68] Godwin CM, Cotner JB. Stoichiometric flexibility in diverse aquatic heterotrophic bacteria is coupled to differences in cellular phosphorus quotas. Frontiers in microbiology. 2015;6:159 10.3389/fmicb.2015.00159 25774154 PMC4343017

[69] Santos-Beneit F. The Pho regulon: a huge regulatory network in bacteria. Frontiers in microbiology. 2015;6:402 10.3389/fmicb.2015.00402 25983732 PMC4415409

[70] Krol E, Becker A. Global transcriptional analysis of the phosphate starvation response in *Sinorhizobium meliloti* strains 1021 and 2011. Molecular Genetics and Genomics. 2004;272(1):1–17. 10.1007/s00438-004-1030-8 15221452

[71] Li Y, Zhang Y. PhoU is a persistence switch involved in persister formation and tolerance to multiple antibiotics and stresses in *Escherichia coli*. Antimicrobial agents and chemotherapy. 2007;51(6):2092–9. 10.1128/AAC.00052-07 17420206 PMC1891003

[72] Hoi LT, Voigt B, Jürgen B, Ehrenreich A, Gottschalk G, Evers S, et al The phosphate‐starvation response of *Bacillus licheniformis*. Proteomics. 2006;6(12):3582–601. 10.1002/pmic.200500842 16705752

[73] Yuan Z-C, Zaheer R, Morton R, Finan TM. Genome prediction of PhoB regulated promoters in *Sinorhizobium meliloti* and twelve proteobacteria. Nucleic acids research. 2006;34(9):2686–97. 10.1093/nar/gkl365 16717279 PMC1464414

[74] Chakraborty S, Sivaraman J, Leung KY, Mok Y-K. Two-component PhoB-PhoR regulatory system and ferric uptake regulator sense phosphate and iron to control virulence genes in type III and VI secretion systems of *Edwardsiella tarda*. Journal of Biological Chemistry. 2011;286(45):39417–30. 10.1074/jbc.M111.295188 21953460 PMC3234765

[75] Smith M, Payne J. Expression of periplasmic binding proteins for peptide transport is subject to negative regulation by phosphate limitation in *Escherichia coli*. FEMS microbiology letters. 1992;100(1–3):183–90. 1478454 10.1111/j.1574-6968.1992.tb14038.x

[76] Dowhan W. Molecular genetic approaches to defining lipid function. Journal of lipid research. 2009;50(Supplement):S305–S10. 18980944 10.1194/jlr.R800041-JLR200 PMC2674694

[77] Laganowsky A, Reading E, Allison TM, Ulmschneider MB, Degiacomi MT, Baldwin AJ, et al Membrane proteins bind lipids selectively to modulate their structure and function. Nature. 2014;510(7503):172 10.1038/nature13419 24899312 PMC4087533

[78] Lamarche MG, Wanner BL, Crépin S, Harel J. The phosphate regulon and bacterial virulence: a regulatory network connecting phosphate homeostasis and pathogenesis. FEMS microbiology reviews. 2008;32(3):461–73. 10.1111/j.1574-6976.2008.00101.x 18248418

[79] Liberek K, Marszalek J, Ang D, Georgopoulos C, Zylicz M. *Escherichia coli* DnaJ and GrpE heat shock proteins jointly stimulate ATPase activity of DnaK. Proceedings of the National Academy of Sciences. 1991;88(7):2874–8. 10.1073/pnas.88.7.2874 PMC51342 1826368

[80] Daines DA, Wright LF, Chaffin DO, Rubens CE, Silver RP. NeuD plays a role in the synthesis of sialic acid in *Escherichia coli* K1. FEMS microbiology letters. 2000;189(2):281–4. 10930752 10.1111/j.1574-6968.2000.tb09244.x

[81] Ye RW, Zielinski NA, Chakrabarty AM. Purification and characterization of phosphomannomutase/phosphoglucomutase from *Pseudomonas aeruginosa* involved in biosynthesis of both alginate and lipopolysaccharide. Journal of bacteriology. 1994;176(16):4851–7. 8050998 10.1128/jb.176.16.4851-4857.1994 PMC196319

[82] Malinverni JC, Silhavy TJ. An ABC transport system that maintains lipid asymmetry in the Gram-negative outer membrane. Proceedings of the National Academy of Sciences. 2009;106(19):8009–14. 10.1073/pnas.0903229106 PMC2683108 19383799

[83] Semeniuk A, Sohlenkamp C, Duda K, Hölzl G. A bifunctional glycosyltransferase from *Agrobacterium tumefaciens* synthesizes monoglucosyl and glucuronosyl diacylglycerol under phosphate deprivation. Journal of Biological Chemistry. 2014;289(14):10104–14. 10.1074/jbc.M113.519298 24558041 PMC3974981

[84] Vences‐Guzmán MÁ, Guan Z, Escobedo‐Hinojosa WI, Bermúdez‐Barrientos JR, Geiger O, Sohlenkamp C. Discovery of a bifunctional acyltransferase responsible for ornithine lipid synthesis in *Serratia proteamaculans*. Environmental microbiology. 2015;17(5):1487–96. 10.1111/1462-2920.12562 25040623

[85] Carini P, Van Mooy BA, Thrash JC, White A, Zhao Y, Campbell EO, et al SAR11 lipid renovation in response to phosphate starvation. Proceedings of the National Academy of Sciences. 2015;112(25):7767–72. 10.1073/pnas.1505034112 PMC4485111 26056292

