# Supplementary figures and images for: System-Wide Adaptations of Desulfovibrio alaskensis G20 to Phosphate-Limited Conditions
Source: PLoS One. 2016 Dec 28;11(12):e0168719. doi: 10.1371/journal.pone.0168719 (PMC5193443; doi:10.1371/journal.pone.0168719)

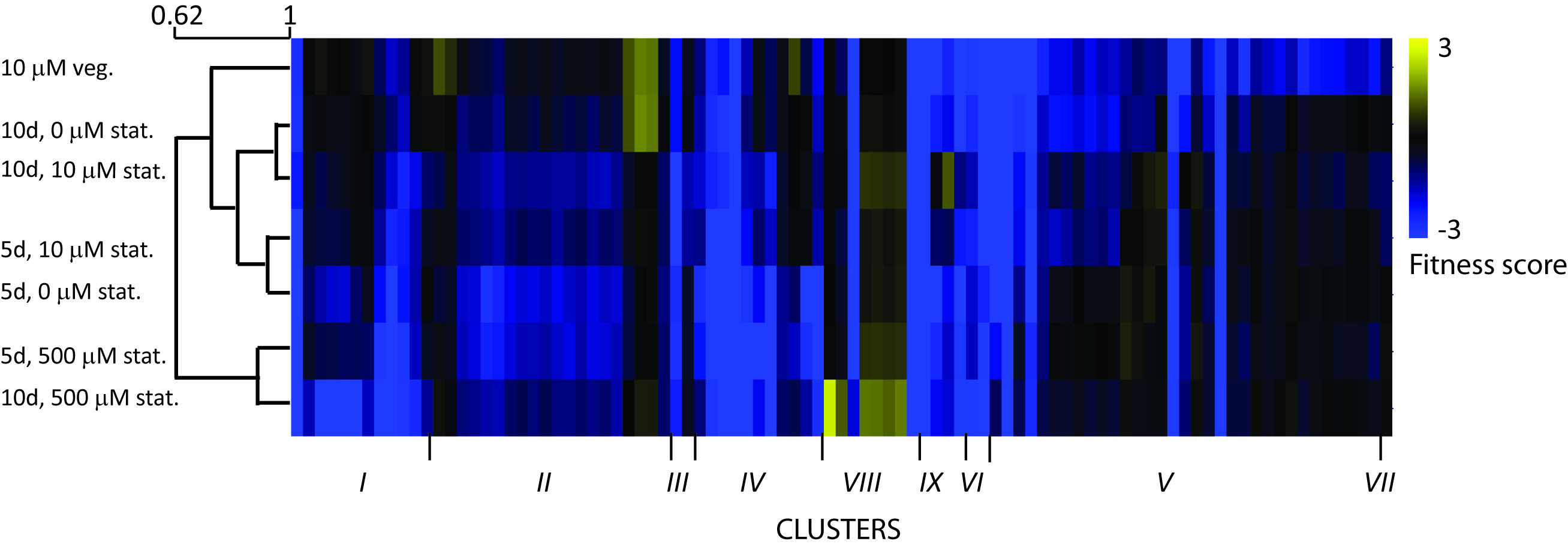

Supplement: S1 Fig — Hierarchical clustering analysis identified nine clusters with distances smaller than 0.5 using the Pearson correlation coefficient as a distance metric and average linkage clustering. “Veg” denotes vegetatively growing pool cultures and “stat” denotes cultures in stationary phase after 5 or 10 days of starvation at 0, 10 or 500 μM initial phosphate in the medium. (JPG) [file pone.0168719.s001.jpg]

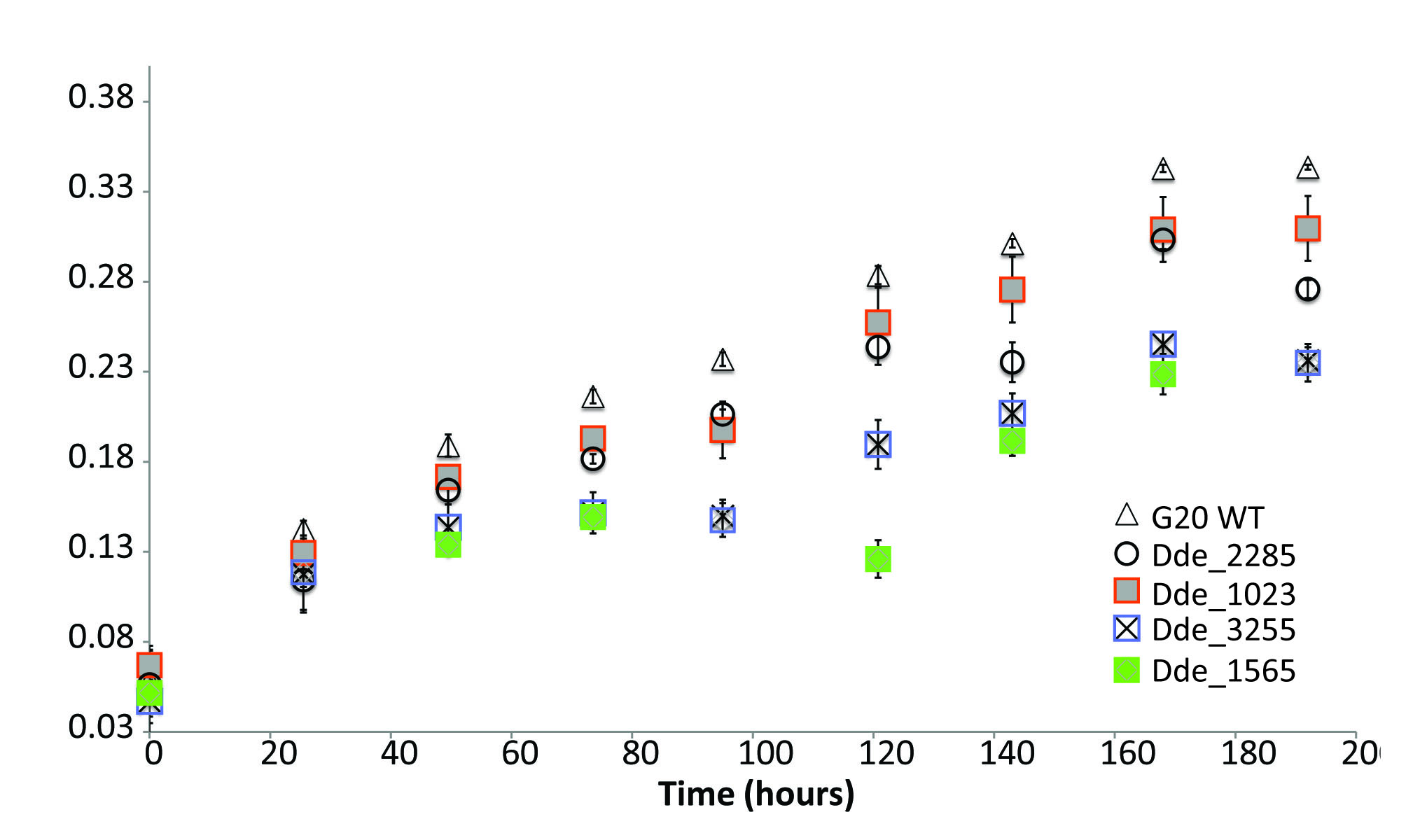

Supplement: S2 Fig — All mutants had fitness defects in pure cultures as well as in mutant pool experiments. G20 WT is the wild-type G20. (JPG) [file pone.0168719.s002.jpg]

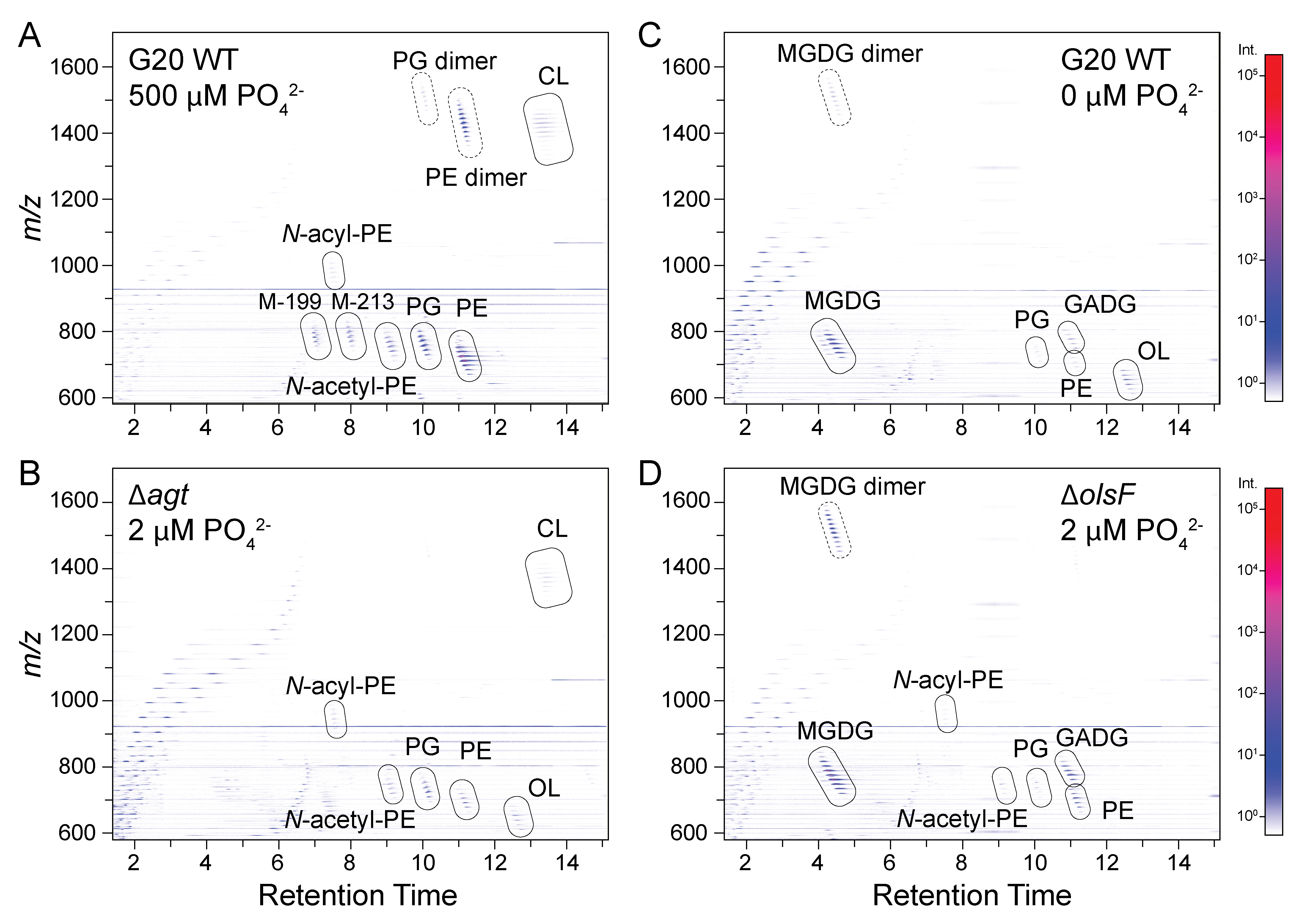

Supplement: S3 Fig — (A) Wild-type G20 in phosphate-replete cultures. (B) Dde_3613 (Δagt) in phosphate-limited culture. (C) Wild-type G20 in phosphate-limited cultures. (D) Dde_3661 (ΔolsF) in phosphate-limited culture. The HPLC-MS chromatograms are depicted as density maps. The x-axis shows the retention time, the y-axis shows m/z, the relative peak intensity is shown in color. PE: phosphatidylethanolamine, PG: phosphatidylglycerol, CL: cardiolipin, OL: ornithine lipids, MGDG: monoglycosyl diacylglycerol, GADG: glycuronic acid diacylglycerol. Dimers form in the ion source during elevated concentrations of analyzed compounds. (TIF) [file pone.0168719.s003.tif]

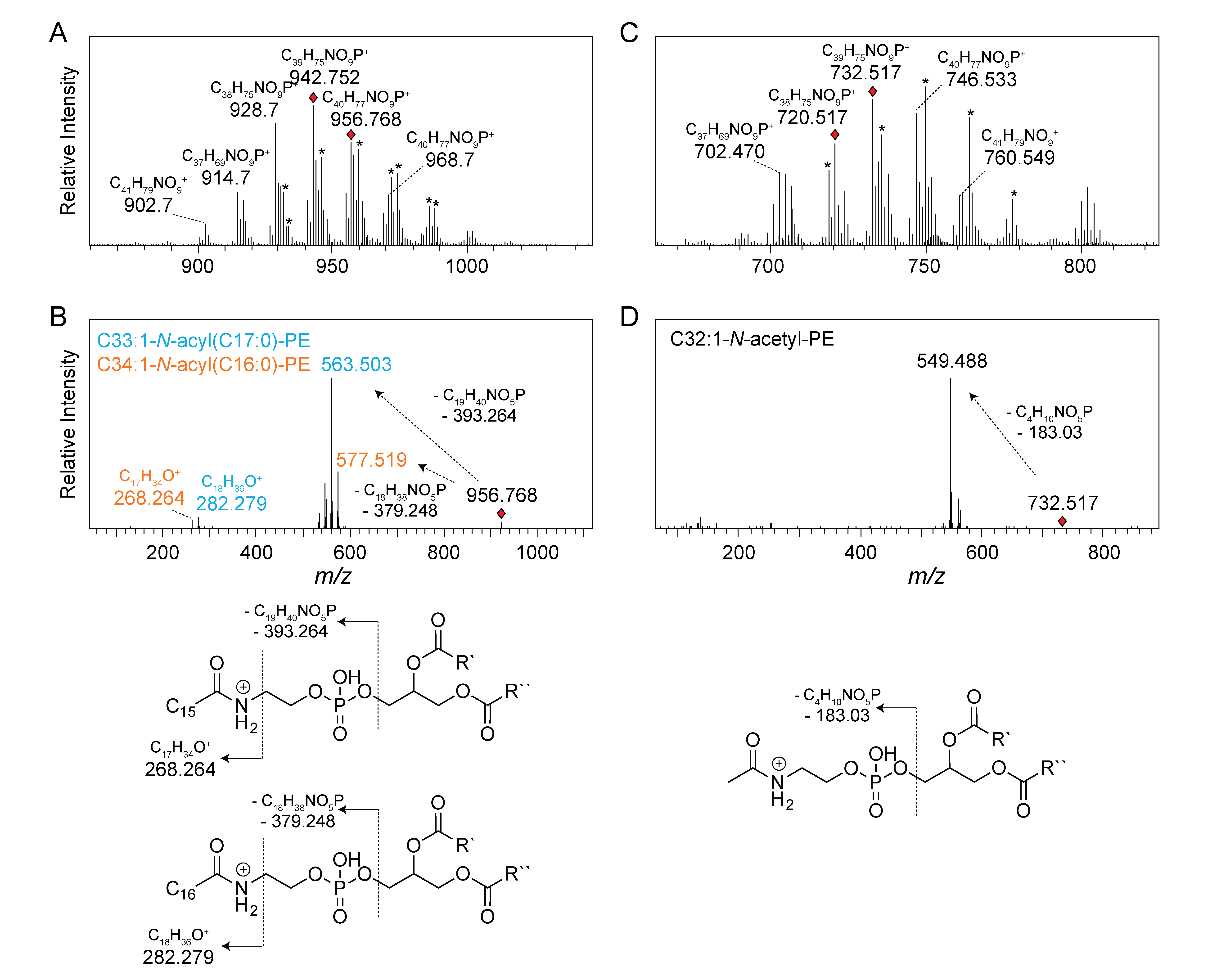

Supplement: S4 Fig — (A) Dominant protonated N-acyl-PE ions detected during full scan (MS1) at 7.5–8 min reflect typical acyl chain heterogeneity. The star (*) denotes complementary ammonium adducts of the protonated molecular ions. (B) High-resolution accurate-mass quadrupole MS2 mass spectrum of N-acyl-PE ions m/z 956.768 and m/z 942.768. The chemical formulas represent neutral losses and products after MS2 fragmentation. As with other multiple-acylated intact polar lipids, a given molecular mass of N-acyl-PE can represent several distinct molecular species. Changes in the acyl chain length can occur both in the diacylglycerol core lipid or the N-acyl-PE head group. For instance, at least two species can explain the ion m/z 956.768, one with a combined diacylglycerol acyl chain length of C32:1 and N-acyl-PE acyl chain length of C17:0 or a combination of C33:1 and C16:0 for diacylglycerol or N-acyl-PE acyl chains, respectively. (C_ Dominant protonated N-acetyl-PE ions detected during full scan (MS1) at 8.5–9.5 min. The star (*) denotes the complementary ammonium adducts to the protonated molecular ions. (D) High-resolution accurate-mass quadrupole MS2 mass spectrum of N-acetyl-PE ions m/z 732.517 and m/z 720.517. The neutral loss of 183.03 observed for these ions indicates the loss of the N-acetyl-PE head group. (TIF) [file pone.0168719.s004.tif]

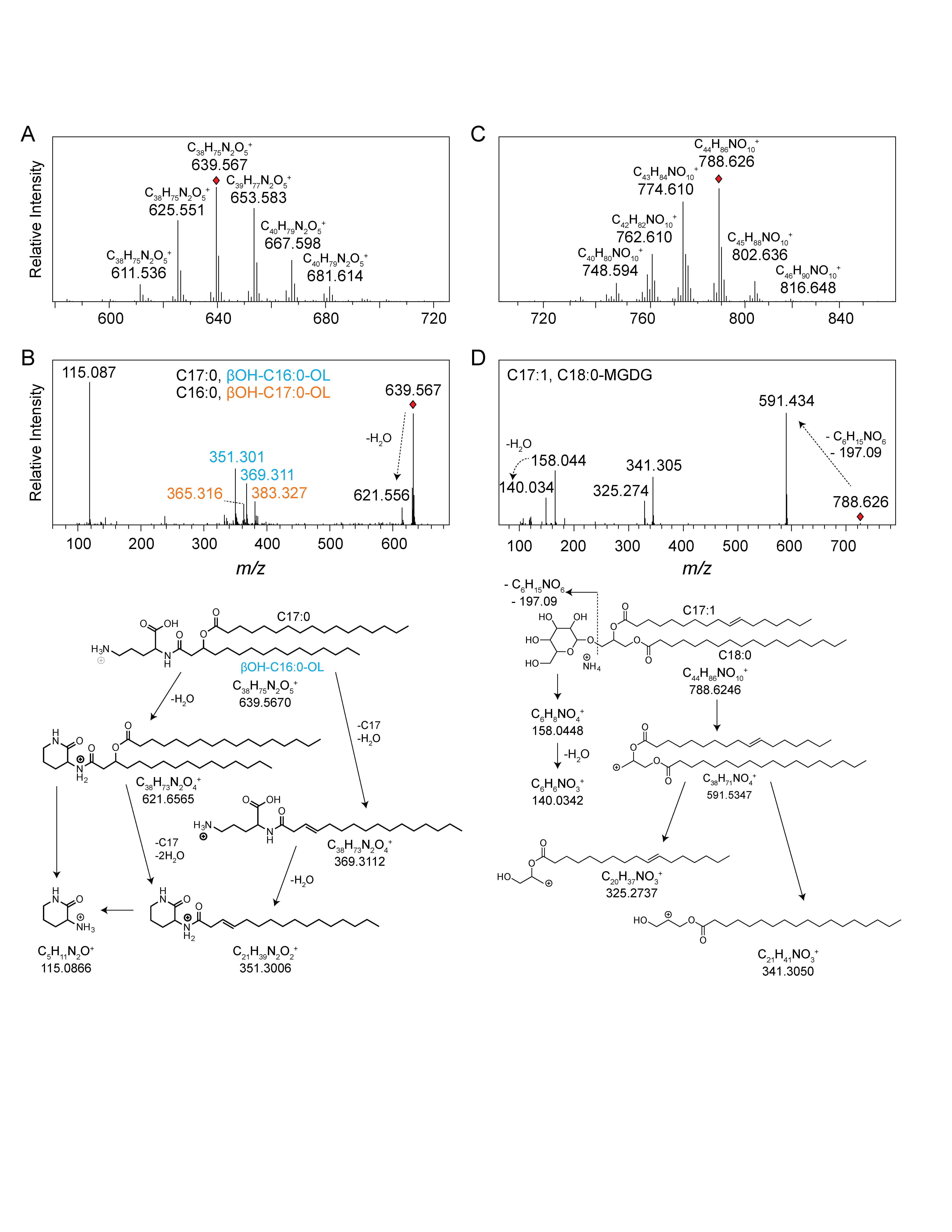

Supplement: S5 Fig — (A) Dominant protonated OL ions detected during full scan (MS1) at 12.5–13 min reflect typical acyl chain heterogeneity. (B) High-resolution accurate-mass quadrupole MS2 mass spectrum of OL ion m/z 639.567, showing major fragments and their chemical formulas. As with other multiple-acylated intact polar lipids, a given molecular mass of OL can represent several distinct molecular species. At least two species can explain the ion m/z 639.567, one with an acyl chain length of C16:0 for the ß-OH fatty acid amide-linked to the ornithine headgroup and a C17:0 acyl chain length for the fatty acid esterified to the hydroxyl group and vice versa. (C) Dominant MGDG ions with ammonium adducts detected during full scan (MS1) at 4–5 min. (D) High-resolution accurate-mass quadrupole MS2 mass spectrum of MGDG ion m/z 788.626. The chemical formulas represent neutral losses and products after MS2 fragmentation. (TIF) [file pone.0168719.s005.tif]

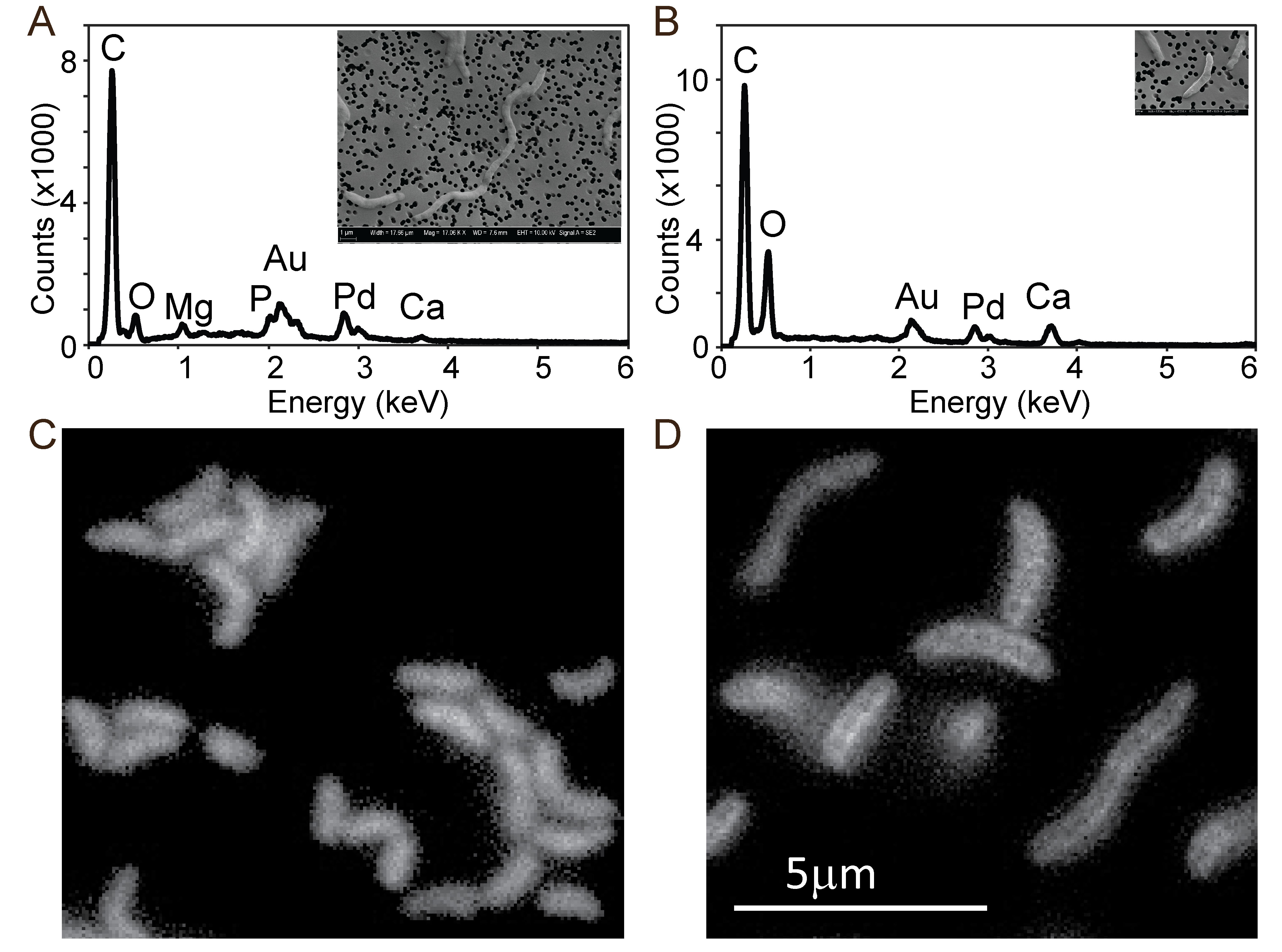

Supplement: S6 Fig — (A) EDS spectrum of cells grown in the phosphate-replete medium contains a phosphorus peak. Inset: Scanning electron micrograph of analyzed cells on a polycarbonate filter. (B) EDS spectrum of cells grown in phosphate-limited medium lack a detectable phosphorus peak. All samples were coated by Au and Pd. (C) Epifluorescence micrograph of cells stained by Nile red after growth in phosphate replete medium. (D) Epifluorescence micrograph of cells stained by Nile red after growth in phosphate limited medium. The scale shown in panel (D) applies to both panels (C) and (D). (JPG) [file pone.0168719.s006.jpg]

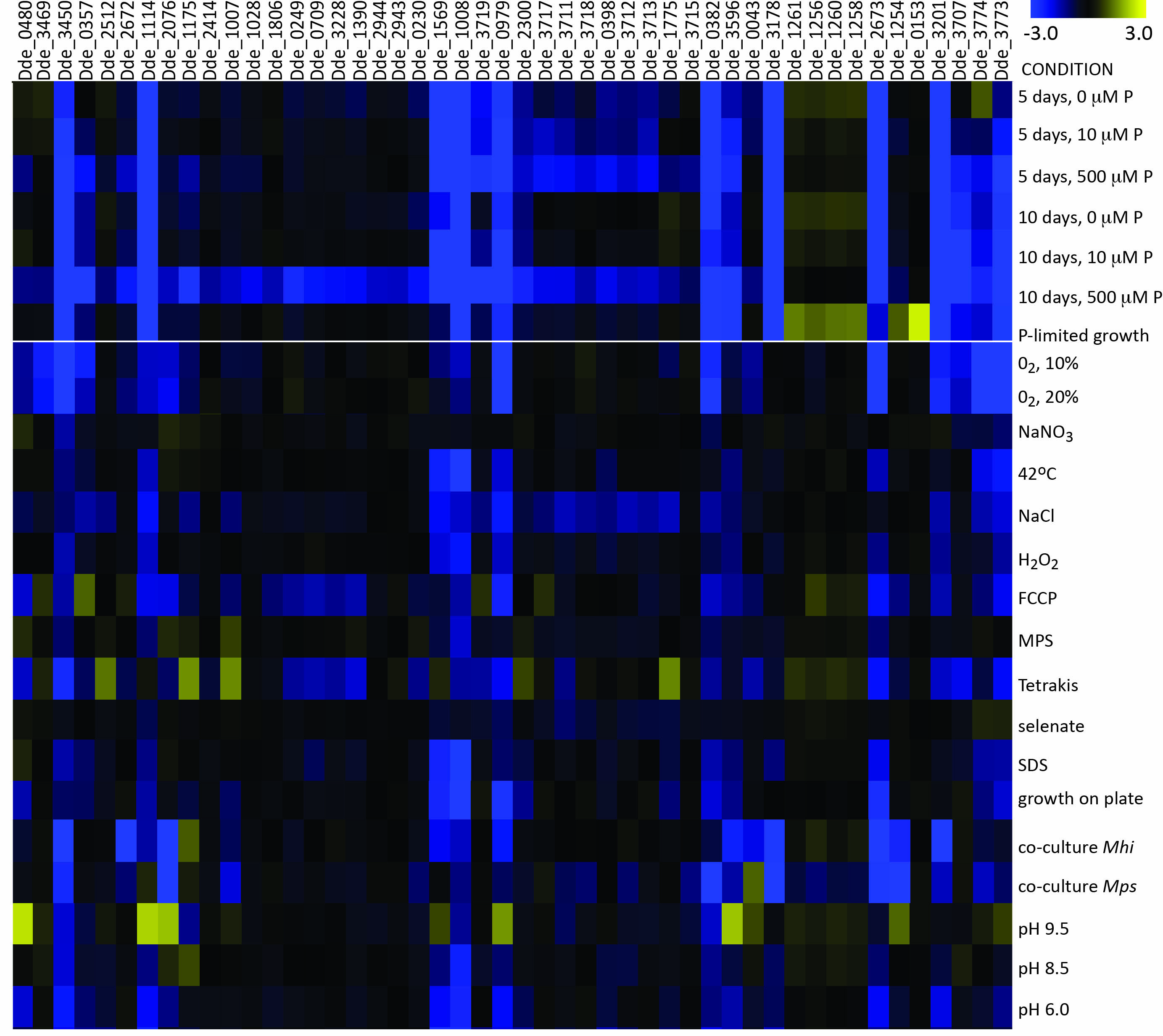

Supplement: S7 Fig — The top seven rows show fitness scores measured in our experiments. Subsequent rows show fitness scores of the same genes during the survival in the presence of 20% and 10% oxygen in the headspace, 150 mM NaNO3, incubation at 42°C, 800 mM NaCl, the daily addition of 200 μM H2O2, 100 μg/ml FCCP, 1 mM monofluorophosphate, 0.1 mM THPS, 0.01 mM selenate, sodium dodecyl sulfate (SDS), growth on lactate/sulfate plates, pyruvate fermentation in co-cultures with Methanospirillum hungatei (Mh) and Methanococcus maripaludis (Mps), respectively, and at pH 9.5, 8.5 and 6. (JPG) [file pone.0168719.s007.jpg]
