# Supplementary material for: System-Wide Adaptations of Desulfovibrio alaskensis G20 to Phosphate-Limited Conditions
Source: PLoS One. 2016 Dec 28;11(12):e0168719. doi: 10.1371/journal.pone.0168719 (PMC5193443; doi:10.1371/journal.pone.0168719)
Supplement: S3 Table — (DOCX) [file pone.0168719.s010.docx]

|  | Vegetative | 5d, 0 | 5d, 10 | 5d, 500 | 10d, 0 | 10d, 10 | 10d, 500 | 15d, 0 | 15d, 10 | 15d, 500 |
| --- | --- | --- | --- | --- | --- | --- | --- | --- | --- | --- |
| Vegetative | 1 | 0.84 | 0.84 | 0.84 | 0.89 | 0.89 | 0.84 | 0.61 | 0.61 | 0.69 |
| 5d, 0 | 0.84 | 1 | 0.95 | 0.89 | 0.84 | 0.84 | 0.89 | 0.61 | 0.61 | 0.69 |
| 5d, 10 | 0.84 | 0.95 | 1 | 0.89 | 0.84 | 0.84 | 0.89 | 0.61 | 0.61 | 0.69 |
| 5d, 500 | 0.84 | 0.89 | 0.89 | 1 | 0.84 | 0.84 | 0.89 | 0.61 | 0.61 | 0.69 |
| 10d, 0 | 0.89 | 0.84 | 0.84 | 0.84 | 1 | 0.95 | 0.84 | 0.61 | 0.61 | 0.69 |
| 10d, 10 | 0.89 | 0.84 | 0.84 | 0.84 | 0.95 | 1 | 0.84 | 0.61 | 0.61 | 0.69 |
| 10d, 500 | 0.84 | 0.89 | 0.89 | 0.89 | 0.84 | 0.84 | 1 | 0.61 | 0.61 | 0.69 |
| 15d, 0 | 0.61 | 0.61 | 0.61 | 0.61 | 0.61 | 0.61 | 0.61 | 1 | 0.73 | 0.61 |
| 15d, 10 | 0.61 | 0.61 | 0.61 | 0.61 | 0.61 | 0.61 | 0.61 | 0.73 | 1 | 0.61 |
| 15d, 500 | 0.69 | 0.69 | 0.69 | 0.69 | 0.69 | 0.69 | 0.69 | 0.61 | 0.61 | 1 |
